# Coding variants identified in patients with diabetes alter PICK1 BAR domain function in insulin granule biogenesis

**DOI:** 10.1172/JCI144904

**Published:** 2022-03-01

**Authors:** Rita C. Andersen, Jan H. Schmidt, Joscha Rombach, Matthew D. Lycas, Nikolaj R. Christensen, Viktor K. Lund, Donnie S. Stapleton, Signe S. Pedersen, Mathias A. Olsen, Mikkel Stoklund, Gith Noes-Holt, Tommas T.E. Nielsen, Mark P. Keller, Anna M. Jansen, Rasmus Herlo, Massimo Pietropaolo, Jens B. Simonsen, Ole Kjærulff, Birgitte Holst, Alan D. Attie, Ulrik Gether, Kenneth L. Madsen

**Affiliations:** 1 Molecular Neuropharmacology and Genetics Laboratory, Department of Neuroscience, Faculty of Health and Medical Sciences, University of Copenhagen, Copenhagen, Denmark.; 2 Department of Biochemistry, University of Wisconsin–Madison, Madison, Wisconsin, USA.; 3 Beta Cell Biology Group, Department of Biomedical Sciences, Faculty of Health and Medical Sciences, University of Copenhagen, Copenhagen, Denmark.; 4 Diabetes Research Center, Division of Diabetes, Endocrinology and Metabolism, Department of Medicine, Baylor College of Medicine, Houston, Texas, USA.; 5 Department of Health Technology, Technical University of Denmark, Kongens Lyngby, Denmark.; 6 Department of Biomedical Sciences, Faculty of Health and Medical Sciences, University of Copenhagen, Copenhagen, Denmark.

**Keywords:** Cell Biology, Metabolism, Beta cells, Insulin, Protein traffic

## Abstract

Bin/amphiphysin/Rvs (BAR) domains are positively charged crescent-shaped modules that mediate curvature of negatively charged lipid membranes during remodeling processes. The BAR domain proteins PICK1, ICA69, and the arfaptins have recently been demonstrated to coordinate the budding and formation of immature secretory granules (ISGs) at the *trans*-Golgi network. Here, we identify 4 coding variants in the PICK1 gene from a whole-exome screening of Danish patients with diabetes that each involve a change in positively charged residues in the PICK1 BAR domain. All 4 coding variants failed to rescue insulin content in INS-1E cells upon knock down of endogenous PICK1. Moreover, 2 variants showed dominant-negative properties. In vitro assays addressing BAR domain function suggested that the coding variants compromised BAR domain function but increased the capacity to cause fission of liposomes. Live confocal microscopy and super-resolution microscopy further revealed that PICK1 resides transiently on ISGs before egress via vesicular budding events. Interestingly, this egress of PICK1 was accelerated in the coding variants. We propose that PICK1 assists in or complements the removal of excess membrane and generic membrane trafficking proteins, and possibly also insulin, from ISGs during the maturation process; and that the coding variants may cause premature budding, possibly explaining their dominant-negative function.

## Graphical abstract

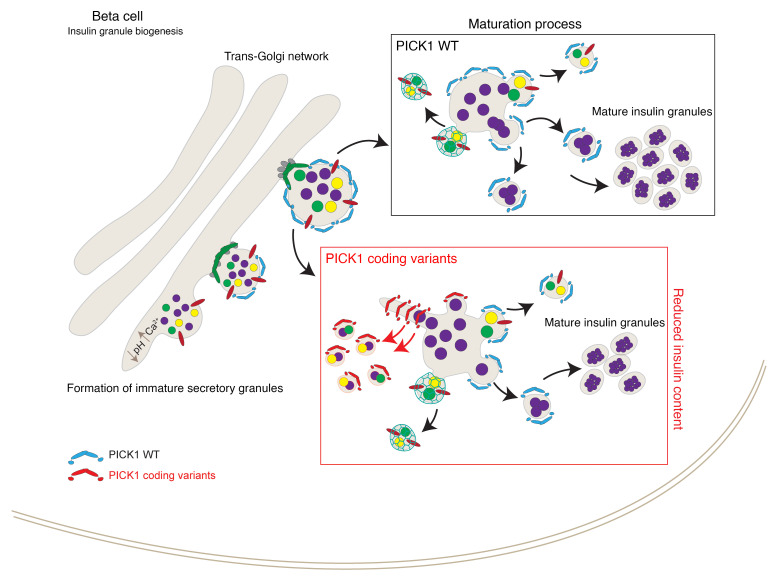

## Introduction

Type 2 diabetes mellitus (T2DM) is a global health problem affecting more than 400 million individuals (1, 2). T2DM is a complex heterogeneous metabolic disease, with an etiology involving a combination of genetic and environmental risk factors. The pathophysiology is characterized by hyperglycemia caused by insulin resistance and impaired insulin secretion, which lead to β cell stress and dysfunction (1). It is therefore not surprising that genes encoding proteins involved in insulin granule biogenesis, such as proprotein convertases and carboxypeptidase E, have been associated with metabolic dysfunction and T2DM (3).

Insulin granules are derived from the regulated secretory pathway (RSP) starting at the *trans*-Golgi network (TGN) and are formed by packaging of proinsulin and other secretory proteins into a nascent bud, followed by a budding process in which immature secretory granules (ISGs) are generated (4, 5). Subsequent processing, including cleavage of proinsulin to insulin followed by condensation in response to acidification and Ca^2+^ influx, leads to development of ISGs into mature secretory granules (MSGs) (6). Moreover, during maturation excess membrane and generic membrane trafficking proteins are removed by budding of vesicles from ISGs, destined for either a constitutive-like secretion, recycling back to the TGN, or to endocytic/lysosomal compartments (7–10). Although the molecular mechanism is unclear, this vesicle budding is thought to be clathrin dependent, as clathrin and adaptor protein 1 (AP-1) are detected on ISGs and budding structures while being absent on MSGs (11–14).

The N-Bin/amphiphysin/Rvs (BAR) domain proteins — comprising islet cell autoantigen 69 (ICA69), protein interacting with C kinase 1 (PICK1), and the arfaptins (collectively named IPA BARs) — together represent a newly discovered group of modulators of the RSP (15). N-BAR domains constitute a distinct class of crescent-shaped dimeric structures that are flanked by amphipathic helices and associate with a high degree of membrane curvature during membrane budding processes via positively charged residues on their concave surface (16–18). Arfaptin 1 stabilizes the nascent budding granules at the TGN by shielding them from the action of ADP–ribosylation factor 1 (ARF1) in a PKD-dependent manner (19); PICK1 as a heterodimer with ICA69 directly assists in the process of budding from the TGN and regulates maturation of insulin granules (20–22). While the physiological importance of arfaptin’s function remains to be addressed, KO studies of PICK1 and ICA69 in mice revealed glucose intolerance as a result of reduced insulin storage and secretion (20–22).

Here, we report coding variants of the IPA N-BAR proteins identified in a cohort of patients with T2DM and focus on 4 coding variants that cause a change in positively charged residues in the PICK1 BAR domain. Functional assessment of the coding variants showed that they failed to rescue the function of PICK1 in insulin granule biogenesis, resulting in reduced insulin storage in INS-1E cells. Two of the coding variants further led to impaired function of WT PICK1 in a dominant-negative manner. The coding variants showed impaired binding to curved lipid membranes, but surprisingly enhanced the ability of PICK1 to facilitate liposome fission. Application of 3D direct stochastic reconstruction microscopy (3D-dSTORM) revealed localization of PICK1 on a subset of insulin granules and further suggested that PICK1 exerts an abscission-like function on insulin granules reminiscent of vesicular budding events. Strikingly, the coding variants accelerated this abscission-like function. We propose that PICK1 egress from ISGs through vesicular budding events during the maturation process assists or complements removal of excess membrane and generic membrane trafficking proteins; and that the coding variants may cause premature budding, possibly explaining their dominant-negative function.

## Results

### Four missense mutations of positively charged residues in the PICK1 BAR domain were identified in a cohort of Danish patients with T2DM.

**Figure 1:**
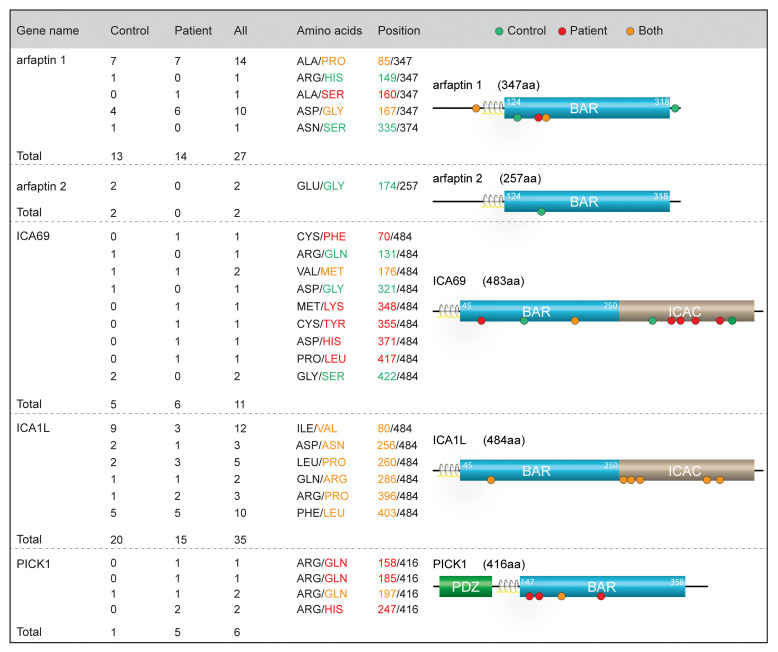
WES of Danish patients with T2DM shows coding variants in the N-BAR domain proteins. Table of heterozygous coding variants identified in the N-BAR domain proteins from a WES of Danish patients with T2DM and a control population. Right: Domain organization of the N-BAR domain proteins, with position of the coding variants. Green, red, and orange dots represent coding variants identified in control participants, patients, and both, respectively.

Whole-exome sequencing (WES) was performed on exomes from 1000 Danish individuals with a combined phenotype of T2DM, moderate adiposity (BMI >27.5 kg/m^2^), and hypertension (systolic/diastolic blood pressure >140/90 mmHg or use of antihypertensive medication); the patients were age matched with 1000 healthy individuals as controls (ctrls) (23). Based on previous indications of the importance of the IPA N-BAR proteins in insulin granule biogenesis, we examined the exomes for coding variants within these proteins (19–22, 24–26). All reported coding variants were heterozygous. In arfaptin 1, we identified 5 coding variants in 27 individuals (13 ctrl/14 patient), and in arfaptin 2 we identified a single coding variant only in 2 individuals in the ctrl group (Figure 1). Furthermore, 9 coding variants in ICA69 (5 ctrl/6 patient) and 6 coding variants in ICA1L (20 ctrl/15 patient) were identified, most of which had alterations in the unstructured C-terminus (Figure 1). Finally, we identified 4 coding variants in PICK1, all of which were present in patients (1 ctrl/5 patient). All 4 missense mutations were in the BAR domain and caused an arginine substitution to either glutamine or histidine: Arg158Gln (R158Q), Arg185Gln (R185Q), Arg197Gln (R197Q), and Arg247His (R247H) (Figure 1). R158Q and R185Q were each identified in 1 individual with T2DM, R247H was detected in 2 individuals with T2DM, while R197Q was identified in 1 individual with T2DM and 1 ctrl individual (Figure 1).

Using AlphaFold2 (27) we assessed putative effects of the PICK1 variants on folding of the BAR domain and on PICK1 homodimerization (Supplemental Figure 1; supplemental material available online with this article; https://doi.org/10.1172/JCI144904DS1). Positively charged residues on the concave side of BAR domains, however, have previously been reported to be essential for binding to negatively charged lipids in membranes (16, 28–30). Thus, we hypothesized that the identified coding variants in the PICK1 BAR domain might compromise the membrane binding and deformation capacity of PICK1, causing impaired insulin granule biogenesis in β cells.

### The coding variants compromise PICK1 clustering in COS-7 cells.

**Figure 2:**
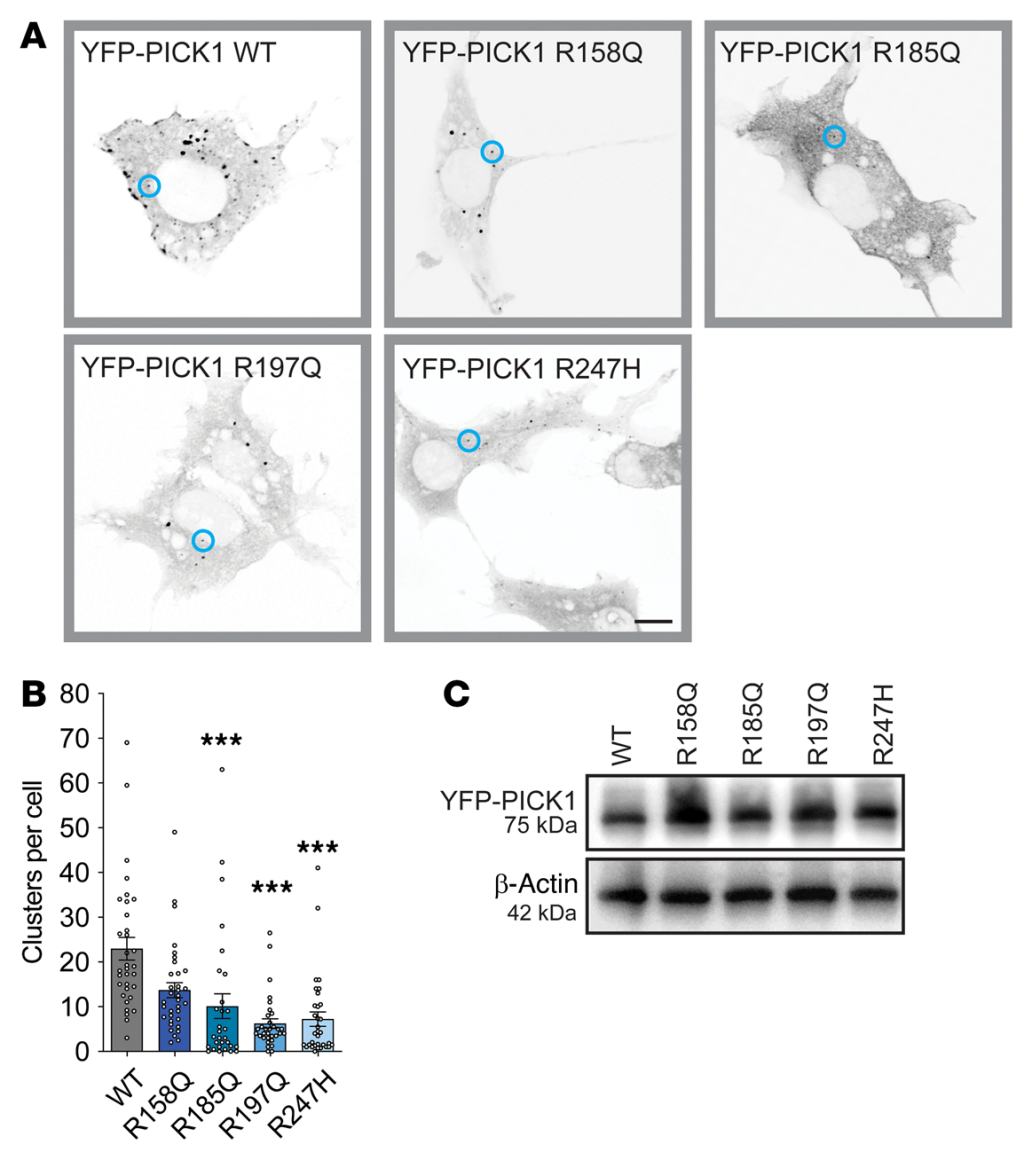
The coding variants display impaired BAR domain function. (**A**) COS-7 cells were transiently transfected with YFP-PICK1 WT and the 4 coding variants. Representative confocal images are shown in inverted gray scale; blue circles represent PICK1 clusters. Scale bar: 10 μm. (**B**) Quantification of clusters per cell. Data are shown as mean ± SEM. Kruskal-Wallis test followed by Dunn’s multiple-comparison test. ***P < 0.001. R158Q (*n =* 43), R185Q (*n =* 32), R197Q (*n =* 31), R247H (*n =* 40) compared with WT (*n =* 41). (**C**) Immunoblotting shows the expression level of YFP-PICK1 WT, R158Q, R185Q, R197Q, and R247H in transiently transfected COS-7 cells.

Whereas N-BAR domain proteins in general show tubular distribution upon overexpression in, for example, COS-7 cells, PICK1 (28–31) — similar to, e.g., endophilin B1 (32) — displays a distinct punctate pattern upon overexpression. This punctate distribution has previously been used to assess BAR domain function, and mutations of positive charges in the BAR domain have been shown to compromise this clustering (28–31). Thus, we examined whether our coding variants affected the clustering propensity of YFP-PICK1. Upon heterologous expression in COS-7 cells, we observed intense clustering of WT PICK1, as described previously (Figure 2, A and B; and refs. 24, 29). Constructs with the coding variants were expressed to the same extent as or to a greater extent than YFP-PICK1 WT (Figure 2C), but all were less prone to clustering than YFP-PICK1 WT, although the difference was not significant for YFP-PICK1 R158Q (Figure 2, A and B).

### The coding variants compromise the function of PICK1, leading to reduced insulin content in INS-1E cells.

**Figure 3:**
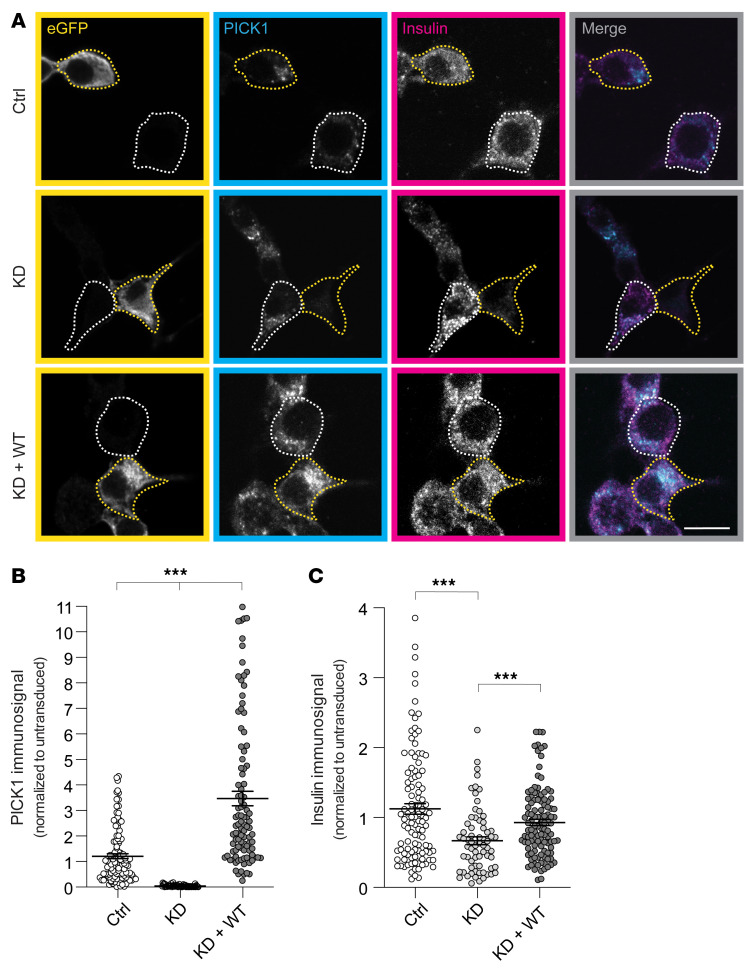
KD of PICK1 in INS-1E cells reduces insulin storage. (**A**) Representative confocal images of INS-1E cells transduced with the lentiviral constructs ctrl, KD, and KD + WT and immunostained for GFP (yellow), PICK1 (cyan), and insulin (magenta). The merged images show PICK1 and insulin immunosignals. Examples of transduced cells (GFP-positive) are outlined with yellow dotted lines, and untransduced cells are outlined with white dotted lines. Scale bar: 10 μm. (**B** and **C**) Quantification of PICK1 and insulin immunosignals from **A**. Data are shown as mean ± SEM. Kruskal-Wallis test followed by Dunn’s multiple-comparison test, ctrl (*n =* 122), KD (*n =* 68), and KD + WT (*n =* 108). ***P < 0.001.

To study the effect of the coding variants in the PICK1 BAR domain on insulin granule biogenesis, we used the insulin-producing INS-1E pancreatic β cell line. We implemented a molecular replacement strategy as previously described (24, 31), using a lentiviral shRNA construct to knock down (KD) endogenous PICK1 expression and reexpress either eGFP alone (referred to herein as KD) or eGFP fused to shRNA-insensitive PICK1 variants — WT or the 4 PICK1 coding variants (referred to as KD + WT, KD + R158Q, KD + R185Q, KD + R197Q, and KD + R247H). A construct expressing eGFP but with deletion of the shRNA was used as a ctrl (Supplemental Figure 2A). INS-1E cells were transduced with the ctrl, KD, and KD + WT constructs and immunostained for PICK1 and insulin (Figure 3A). Quantification of the PICK1 immunosignal in eGFP-positive cells showed a robust decrease in PICK1 expression for KD compared with ctrl, while reexpression of the shRNA-insensitive KD + WT construct increased the PICK1 expression level (Figure 3B). Immunoblotting confirmed KD of endogenous PICK1, despite relatively low transduction efficiency (~30%), and reexpression of eGFP-PICK1 by KD + WT (Supplemental Figure 2, B and C).

Quantification of the insulin immunosignal in eGFP-positive cells showed an approximately 45% reduction compared with ctrl upon KD of PICK1, whereas KD + WT significantly rescued insulin expression, although not fully to the level of the ctrl (Figure 3C). The same pattern was observed using an ELISA (Supplemental Figure 3A), confirming findings of previous studies in both INS-1E cells and isolated islets on the role of PICK1 in insulin granule biogenesis (20, 22, 24).

**Figure 4:**
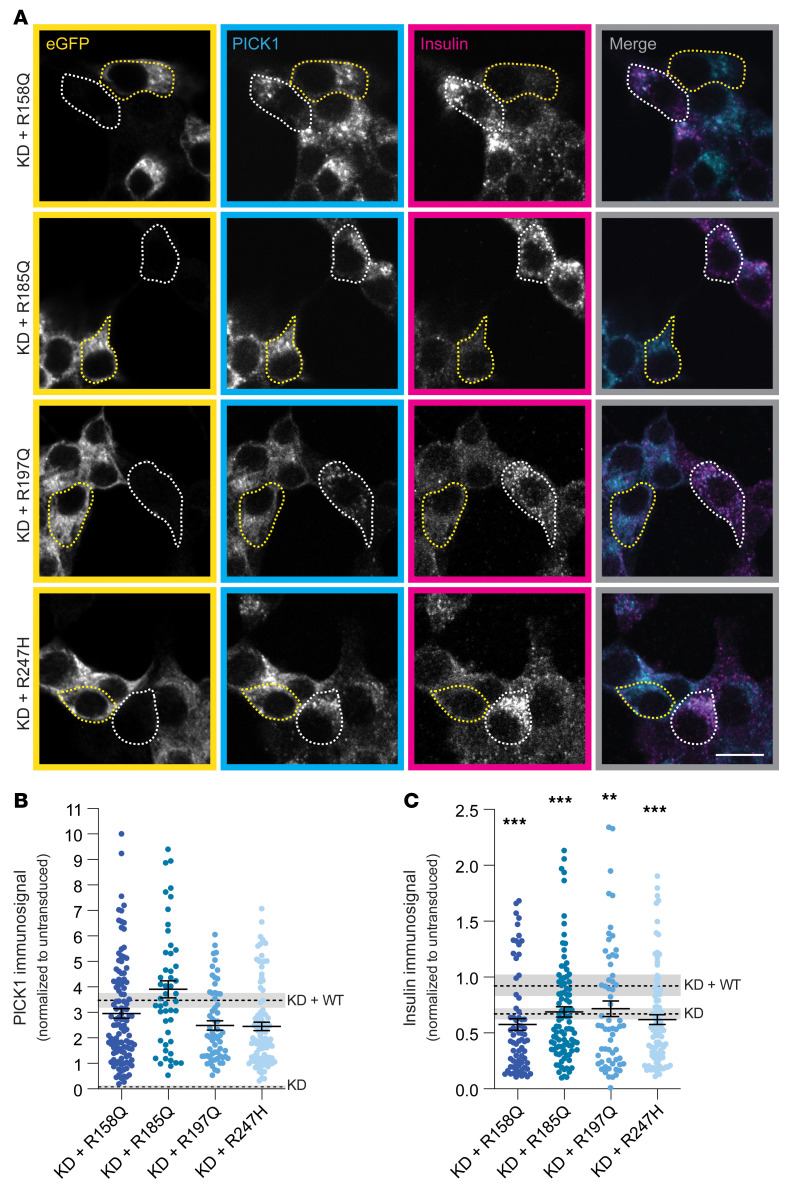
Coding variants in the BAR domain compromise PICK1’s function in insulin storage in INS-1E cells. (**A**) Representative confocal images of INS-1E cells transduced with lentiviral constructs reexpressing PICK1 with each of the 4 coding variants and immunostained for GFP (yellow), PICK1 (cyan), and insulin (magenta). The merged images show PICK1 and insulin immunosignals. Examples of transduced cells (GFP-positive) are outlined with yellow dotted lines, and untransduced cells are outlined with white dotted lines. Scale bar: 10 μm. (**B** and **C**) Quantification of PICK1 and insulin immunosignals from **A**. The dashed lines show values for KD and KD + WT PICK1 and insulin immunosignals (mean ± SEM) from Figure 3, B and C. Data are shown as mean ± SEM. **P < 0.01, ***P < 0.001, KD + R158Q (*n =* 112), KD + R185Q (*n =* 50), KD + R197Q (*n =* 64), KD + R247H (*n =* 102) compared with KD + WT (*n =* 108); Kruskal-Wallis test followed by Dunn’s multiple-comparison test.

To examine whether the PICK1 coding variants could rescue the insulin immunosignal upon KD of endogenous PICK1, we quantified insulin expression from INS-1E cells transduced with the corresponding lentiviral constructs (Figure 4A). The PICK1 immunosignals for the coding variants, similar to KD + WT, were increased compared with untransduced cells (indicated by a value of 1; Figure 4B).

Nonetheless, all 4 PICK1 coding variants failed to rescue insulin expression, with significant reductions (~20%–40%) compared with KD + WT (Figure 4C). The failure to rescue the insulin level was confirmed by ELISA, although the effect on insulin levels was slightly less pronounced, since not all cells were transduced (Supplemental Figure 3B).

The decreased insulin content upon KD of PICK1 could be a consequence of unprocessed proinsulin, as previously reported (20, 22). However, using ELISA we observed no differences in proinsulin content after KD of PICK1 compared with ctrl or KD + WT (Supplemental Figure 2C), nor did we observe a significant change in proinsulin content after reexpression of the 4 PICK1 coding variants (Supplemental Figure 3, C and D).

We next assessed the localization of eGFP-PICK1 and variants to the early secretory pathway, as evaluated by colocalization with TGN38, syntaxin 6, and insulin (Supplemental Figure 4, A–F). We found that localization of the coding variants was quite similar to that of PICK1 WT, which was somewhat surprising given the reduced clustering in COS-7 cells. Also, colocalization with the heterodimeric BAR domain partner ICA69 was unaltered for the coding variants (Supplemental Figure 4, G and H). Using AlphaFold2 (27), we further assessed effects of the variants on heterodimerization with ICA69, but despite the finding that 2 of the involved residues led to putative interactions between PICK1 and ICA69 monomers (R158Q and R197Q), no changes to the overall structure of the heterodimer were seen (Supplemental Figure 5, A and B). In accordance with these findings, all variants coimmunoprecipitated endogenous ICA69 from INS-1E cells at levels similar to eGFP-PICK1 WT (Supplemental Figure 5, C and D).

### Expression of R158Q and R247H in INS-1E cells decreased the insulin immunosignal in a dominant-negative fashion.

**Figure 5:**
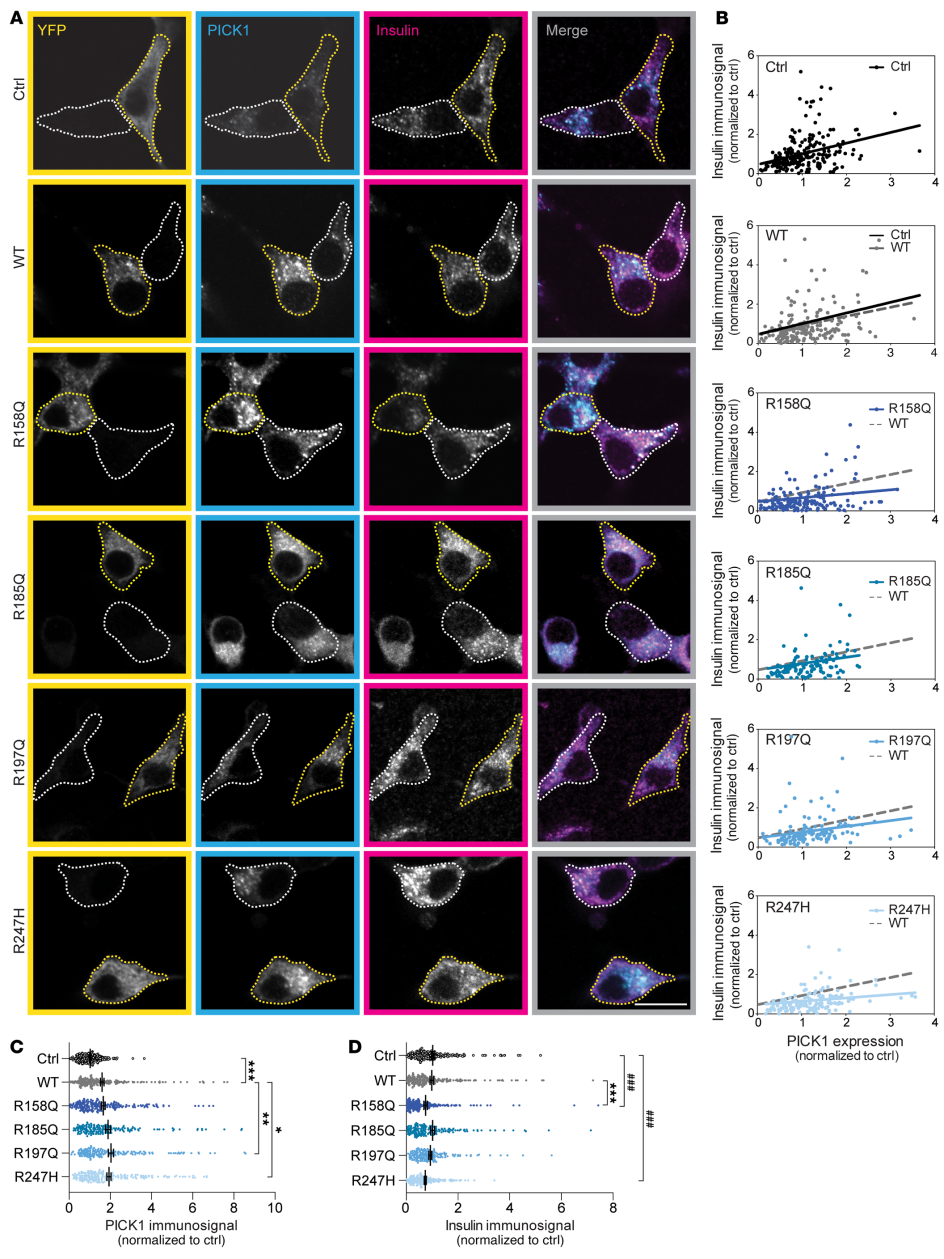
Expression of PICK1-R158Q and PICK1-R247H in INS-1E cells reduces insulin storage. INS-1E cells were transiently transfected with YFP alone (ctrl) or YFP fused to PICK1 WT, R158Q, R185Q, R197Q, and R247H. (**A**) Representative confocal images of INS-1E cells immunostained for YFP (yellow), PICK1 (cyan), and insulin (magenta). Merged images show PICK1 and insulin immunosignals. Examples of transfected cells (YFP-positive) are outlined with yellow dotted lines, and untransfected cells are outlined with white dotted lines. Scale bar: 10 μm. (**B**) Quantified insulin and PICK1 immunosignals per cell correlate and can be fitted with a linear regression. The gray dotted line represents the linear regression for PICK1 WT. Expression of PICK1, R158Q, or R247H reduces the slope of the correlation line. (**C**) Quantification of the PICK1 immunosignal. R158Q (*n =* 159), R185Q (*n =* 119), R197Q (*n =* 125), R247H (*n =* 140), ctrl (*n* = 227) compared with WT (*n =* 188), with Kruskal-Wallis test followed by Dunn’s multiple-comparison test. Data are shown as mean ± SEM. (**D**) Quantification of the insulin immunosignal. *P < 0.05, **P < 0.01, ***P < 0.001, PICK1 coding variants compared with WT; ^###^*P <* 0.001, variants compared with ctrl; Kruskal-Wallis test followed by Dunn’s multiple-comparison test.

Since all 5 patients identified as having the coding variants in PICK1 were heterozygous, we next assessed the putative dominant-negative role of the mutations. To this end, we overexpressed the PICK1 coding variants along with endogenous PICK1 by transiently transfecting INS-1E cells with YFP-PICK1 WT or one of the 4 PICK1 coding variants and immunostained for PICK1 and insulin (Figure 5A). As expected, the PICK1 immunosignal in INS-1E cells overexpressing YFP-PICK1 WT or the 4 coding variants was increased compared with ctrl-transfected cells (indicated by a value of 1; Figure 5C). Interestingly, correlations of the quantified insulin immunosignal versus the corresponding PICK1 immunosignal of individual cells suggested a decreased slope for R158Q (*a* = 0.201 ± 0.097) and R247H (*a* = 0.171 ± 0.058) in particular compared with PICK1 WT (*a* = 0.46 ± 0.11; Figure 5B), indicating that the 2 PICK1 coding variants have a negative effect on insulin content. Quantification of the total insulin immunosignal revealed a significant reduction (by ~25%) for R158Q and R247H compared with PICK1 WT and/or ctrl (Figure 5D). Overexpression of neither R185Q nor R197Q caused changes in the correlation between insulin and PICK1 or the total insulin immunosignal compared with ctrl or PICK1 WT (Figure 5, B–D). These data suggest that R158Q and R247H not only have reduced functionality, but also can exert a dominant-negative effect to suppress the function of PICK1 WT.

**Figure 6:**
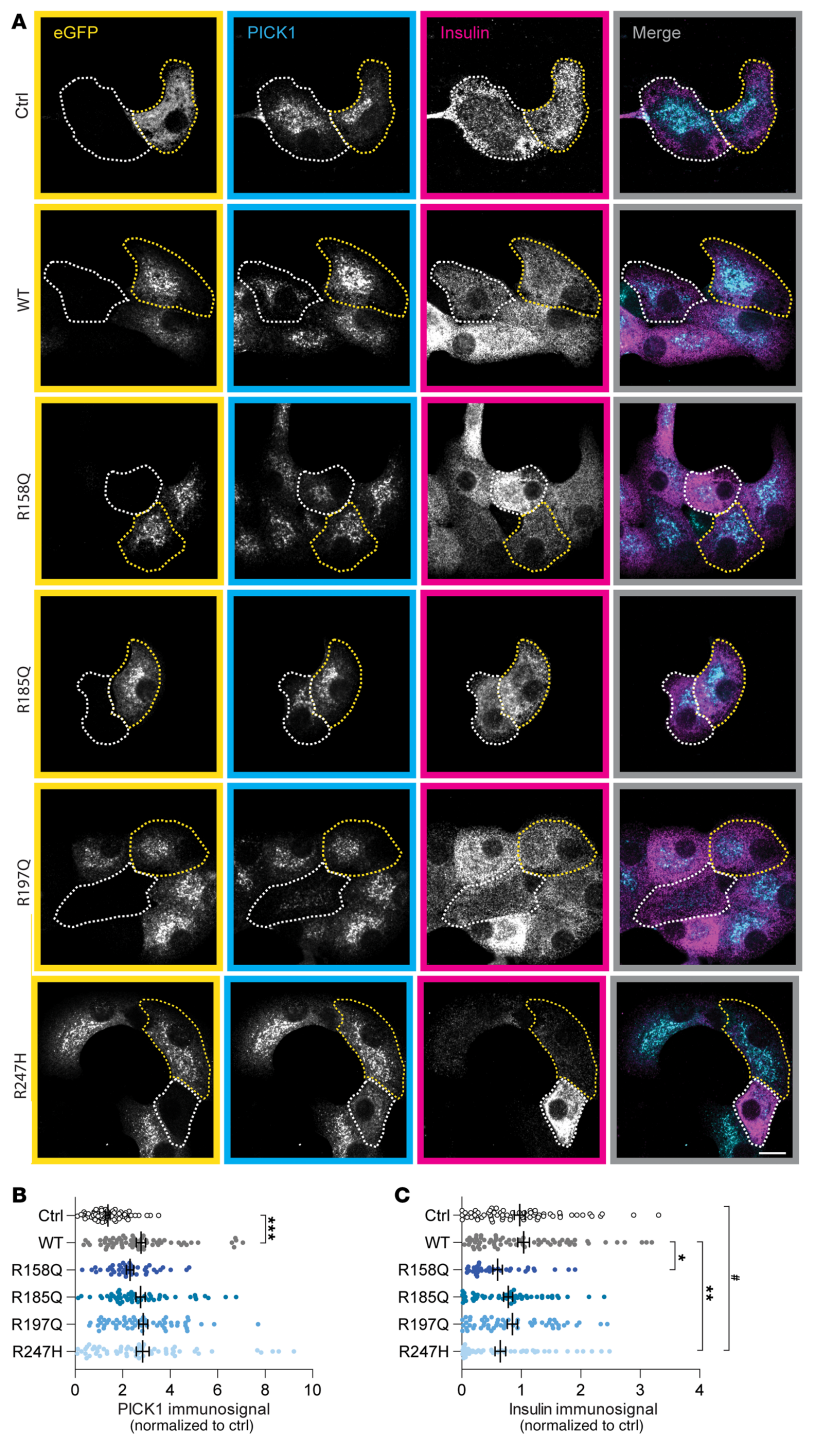
Expression of PICK1-R158Q and PICK1-R247H in mouse β cells reduces insulin storage. (**A**) Representative confocal images of mouse β cells transduced with the lentiviral constructs ctrl, eGFP-PICK1 WT, or the coding variants and immunostained for GFP (yellow), PICK1 (cyan), and insulin (magenta). The merged images show PICK1 and insulin immunosignals. Examples of transduced cells (eGFP-positive) are outlined with yellow dotted lines, and untransduced cells are outlined with white dotted lines. Scale bar: 10 μm. (**B**) Quantification of the PICK1 immunosignal. R158Q (*n =* 42), R185Q (*n =* 58), R197Q (*n =* 58), R247H (*n =* 63), and ctrl (*n =* 69) compared with WT (*n =* 70); Kruskal-Wallis test followed by Dunn’s multiple-comparison test. Data are shown as mean ± SEM. (**C**) Quantification of the insulin immunosignal. *P < 0.05, **P < 0.01, ***P < 0.001, PICK1 coding variants compared with WT; ^#^*P <* 0.05, variants compared with ctrl; Kruskal-Wallis test followed by Dunn’s multiple-comparison test.

Given the large storage capacity and modest turnover rates of insulin in pancreatic β cells, we next addressed whether the coding variants would manifest in dominant-negative effects on insulin storage in primary mouse β cells. To this end, we isolated and trypsinized pancreatic islets from C57BL/6NRj mice to obtain single β cells, before transduction with the lentiviral constructs encoding eGFP-PICK1 WT and the variants. Because the shRNA was designed to target rat PICK1 (31), it was ineffective in KD of PICK1 in primary β cells from mice (Supplemental Figure 6). eGFP-PICK1 immunosignal was clearly visible in numerous cells and significantly increased the total PICK1 immunosignal in these compared with neighboring cells, as determined using a PICK1 antibody recognizing both endogenous and exogenous PICK1 (Figure 6, A and B). Expression of both eGFP-PICK1 R158Q and R247H significantly reduced the insulin immunosignal compared with eGFP-PICK1 WT, whereas overexpression of neither R185Q nor R197Q caused changes in insulin levels (Figure 6, A–C), thereby recapitulating the findings from the INS-1E cell line (Figure 5).

### The coding variants alter PICK1 fission capacity.

**Figure 7:**
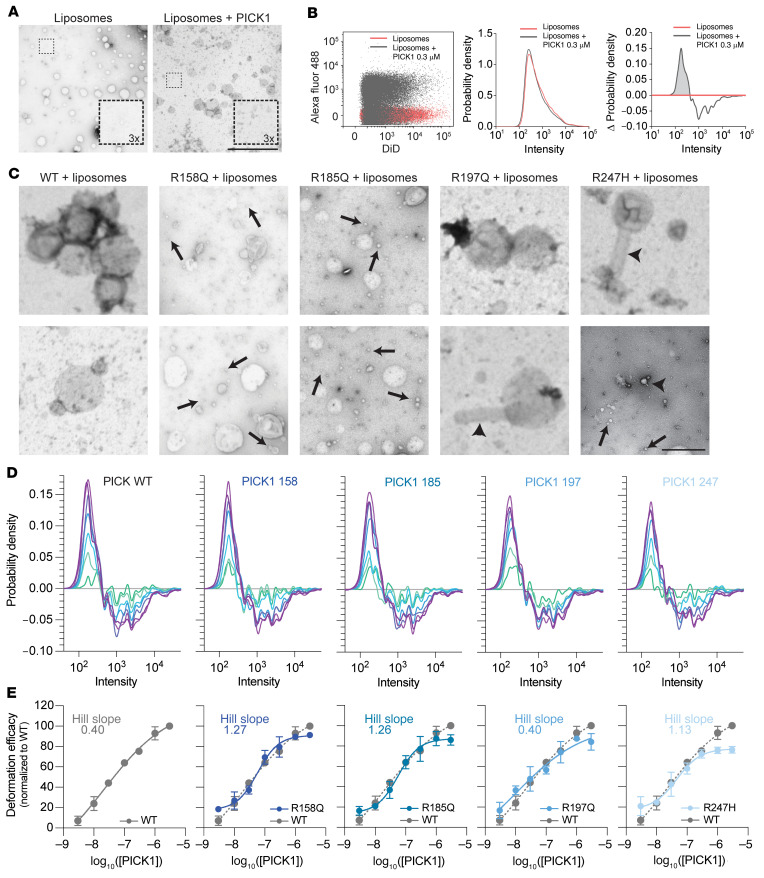
The PICK1 coding variants show increased fission efficacy at lower concentration and altered liposome deformation. (**A**) Representative negative-stain TEM images. Left: Liposomes alone. Right: Liposomes incubated with 0.3 μM PICK1. Scale bar: 2 μm. (**B**) Left: Representative 2-parameter scatter plot of primary data output from the flow cytometer showing fluorescence intensities (in AU) of Alex Fluor 488 (PICK1) versus DiD (liposomes) for samples containing liposomes (red) or liposomes incubated for 1 hour with 0.3 μM AF488 PICK1 (gray). Center: Probability density distribution of DiD intensities extracted from the scatter plot (left), showing the size distribution of DiD-labeled liposomes prior to incubation with PICK1 (red) and liposomes incubated for 1 hour with 0.3 μM Alexa Fluor 488–PICK1 (gray). Right: Change in density distribution after 1 hour of incubation (gray) (kernel density estimations of DiD fluorescence normalized to control). (**C**) Representative negative-stain TEM images of liposomes incubated with PICK1 WT and the PICK1 coding variants for 1 hour. Arrowheads indicate tubular structures, while arrows point to small liposome structures. Scale bar: 1 μm. (**D**) Representative results of flow cytometry experiments show the changes in density distribution of liposomes (kernel density estimations of DiD fluorescence normalized to control) upon incubation (1 hour) with PICK1 WT and the PICK1 coding variants at a concentration range of 0.003–3 μM as a measure of liposome fission efficacy. (**E**) Liposome fission efficacy quantified as the area under the curve of normalized kernel density estimations of DiD fluorescence for a concentration range from 0.003 to 3 μM for PICK1 and the PICK1 coding variants. The points were fitted to a sigmoidal standard curve: the respective Hill slopes are shown in each plot.

Recent studies have demonstrated that BAR domain proteins have potent fission capacity, challenging the current consensus that they are general stabilizers of tubular membrane structures (33, 34). To directly visualize such putative membrane deformation and fission capacity, we incubated liposomes with purified PICK1 WT and observed the resulting structures by transmission electron microscopy (TEM).The images revealed frequent small- to medium-sized adherent liposomes (50–200 nm), as well as numerous small structures (>50 nm), none of which were observed in the absence of PICK1 (Figure 7A). This suggests the propensity of PICK1 to elicit abscission of large liposomes into small- to medium-sized liposomes, rather than inducing tubulation, as seen for most N-BAR domains (16). To quantitatively assess this deformation, we applied a single liposome deformation (SLiD) assay adapted to high-throughput analysis by flow cytometry. We applied purified PICK1 to small unilamellar vesicles (SUVs) prepared from bovine brain extract (Folch fraction 1, ~2.5 μg/mL) and labeled with lipophilic dye (DiD); this was followed by incubation for 1 hour. The distribution of fluorescence intensities of single events, representing individual liposomes, was plotted using kernel density estimates (Figure 7B, red line) and matched previously reported distributions, with a peak at log 2.4 corresponding to liposome diameters between 50 and 100 nm (35). Upon incubation with PICK1, a small but reproducible shift in the distribution toward lower intensities was observed (Figure 7B). This change in distribution was visualized by normalization to the original liposome distribution, which showed an increase in the frequency of liposomes with intensities from log 2 to log 2.6, at the expense of liposomes with intensities greater than log 2.6 (Figure 7B), indicative of an overall process of liposome fission.

We next addressed the fission capacity of the PICK1 coding variants. All variants purified equally well to PICK1 WT, and PDZ domain function was unaltered, attesting to the overall integrity of the protein (Supplemental Figure 7, A–C). Whereas PICK1 WT primarily induced the formation of the adherent structures described above, among the variants, such structures were observed only in the case of R197Q. Moreover, PICK1 induced more discrete abscissions, and this was mimicked by R185Q but not the other variants (Figure 7C). Finally, all the variants induced structures with a high degree of curvature — nascent buds (R158Q), small-sized liposomes (R185Q), and tubular membrane structures (R197Q and R247H) — akin to the structures observed with other N-BAR domains.

To quantitatively compare the deformation capacity of the coding variant to PICK1 WT, we assessed dose-dependent (3–3000 nM PICK1) deformation using the SLiD assay. For PICK1 WT, this revealed a clear dose dependence of fission activity (represented as area under the curve), with a half-maximum effect at 21 nM PICK1 and a low Hill coefficient (0.40; Figure 7, D and E). R197Q displayed a concentration dependence similar to that of PICK1 WT (EC_50_ = 11 nM) and similarly low Hill coefficient (i.e., 0.4), whereas R158Q, R185Q, and R247H displayed Hill coefficients greater than 1, as well as slightly higher concentrations to reach half-maximal binding (58, 57, and 46 nM, respectively) (Figure 7, D and E). Moreover, all coding variants showed a higher degree of deformation at low concentrations and a lower degree of deformation at high concentrations. The overall shapes of the curves showing normalized density changes were identical for PICK1 WT and the coding variants (Figure 7D), implying that the size dependence of fission was not affected by the coding variants. In summary, the coding variants induced structures with a higher level of curvature than did PICK1 WT and displayed altered concentration dependence, with R197Q being least different from PICK1 WT.

### PICK1 resides transiently on insulin ISGs before budding off during maturation.

**Figure 8:**
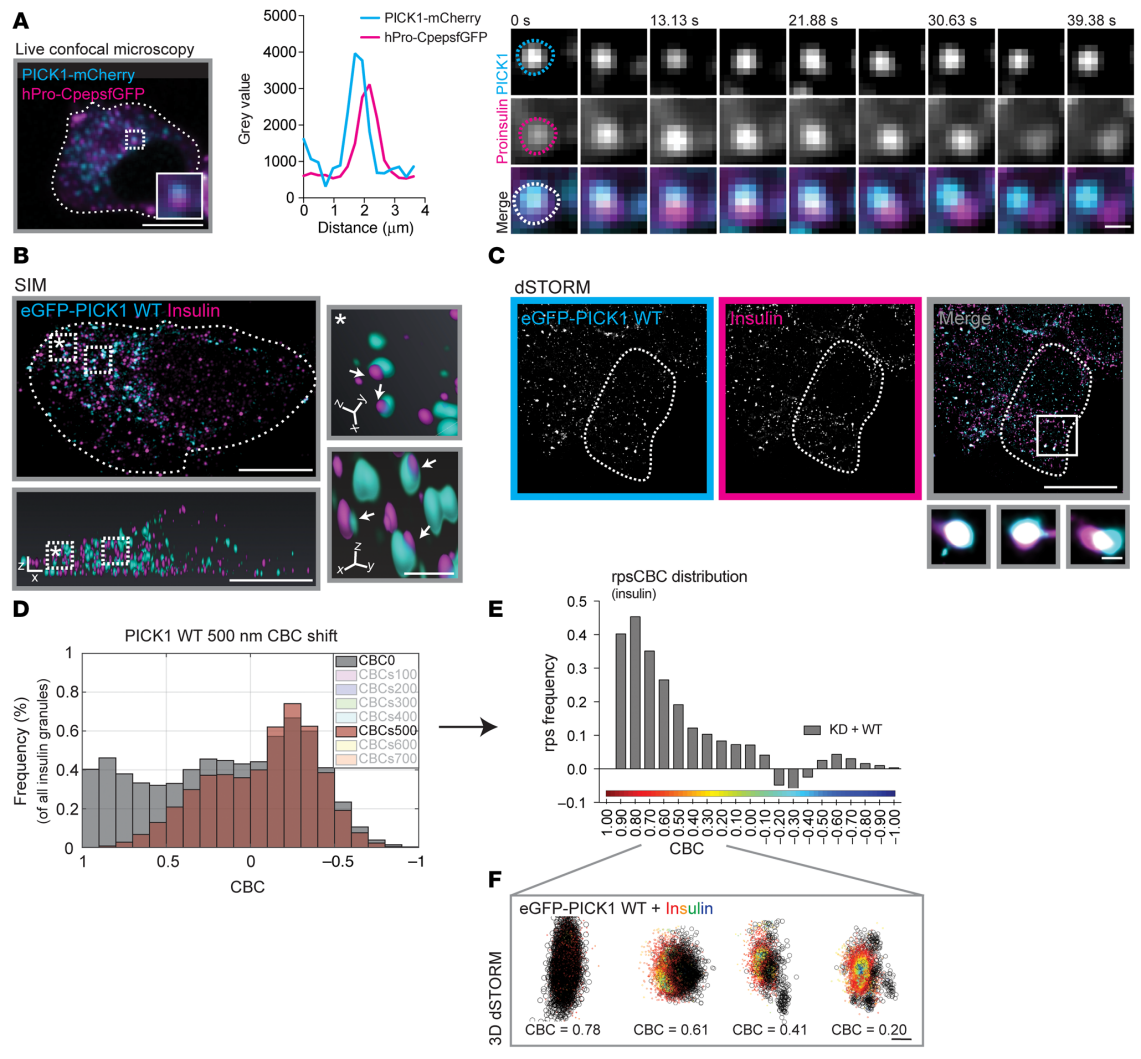
PICK1 resides transiently on insulin ISGs before budding off. (**A**) GRINCH cells were transiently transfected with PICK1-mCherry. Representative GRINCH cell shows a colocalized hPro-CpepsfGFP (magenta) and PICK1-mCherry (cyan) cluster, indicated by the dotted square, with the inset (30% zoom) highlighting the overlap. Scale bar: 10 μm. Right: Profile plot from the inset and time-lapse images of the merged *Z*-stack (500 nm) during steady-state conditions (11 mM glucose). Time is in seconds. Upper 2 rows present a PICK1-mCherry cluster and hPro-CpepsfGFP cluster, respectively, both shown in gray scale. The third row shows merge images. Time is in seconds. Scale bar: 1 μm. (**B** and **C**) INS-1E cells transduced with KD + WT and immunostained for eGFP-PICK1 (cyan) and insulin (magenta). (**B**) Representative SIM image of INS-1E cells. Bottom: 3D reconstruction. Scale bar: 5 μm. Right: Insets with higher-magnification images of overlapping PICK1 and insulin granules (arrows) in 3D. Scale bar: 500 nm. (**C**) Representative dSTORM image of INS-1E cells. Scale bar: 5 μm. Bottom: Insets with higher-magnification images of overlapping PICK1 and insulin granules. Scale bar: 250 nm. (**D**) Insulin CBC shift analysis. PICK1 clusters shifted +500 nm in the *x* direction, and the CBC distribution of the insulin granules was recalculated (brown) and overlaid on the original CBC distribution (gray). (**E**) The difference in CBC between the original from **B** (gray) and the +500 nm shift (brown). We refer to this as rpsCBC distribution. Note that many points are not assigned CBC values (NA) when shifted. *n =* 5 individual experiments. (**F**) The 3D images display distinctive colocalized clusters of insulin (colored by CBC scale) and eGFP-PICK1 (black), ordered by CBC values ranging from 0.78 to 0.20 and indicative of PICK1 fission from insulin granules. Scale bar: 200 nm.

Next, we assessed whether PICK1-dependent abscission of liposomes might relate to its function in dense-core vesicle biogenesis. To evaluate the dynamic association of PICK1 with secretory granules in living cells, we took advantage of the glucose-responsive, insulin-secreting, C-peptide–modified human proinsulin (GRINCH) INS-1 cell line, which stably expresses hPro-CpepsfGFP (36). Live confocal microscopy of GRINCH cells transiently transfected with PICK1-mCherry demonstrated partial overlap of the signal from PICK1-mCherry and hPro-CpepsfGFP (Figure 8A and Supplemental Figure 8), which is in agreement with our immunostaining results (Supplemental Figure 4, A and B) and previous live microscopy studies with PICK1 and phogrin (20). By following the dynamics of individual puncta of PICK1-mCherry and hPro-CpepsfGFP clusters in GRINCH cells, we often observed separation of the colors over time, indicating that the association of PICK1 with insulin granules was of an extended but ultimately transient nature (Figure 8A, Supplemental Figure 8, and Supplemental Video 1).

Interpretation of live microscopy can be obscured by rotation and overlay in the *z* axis within confocal slices, so to further examine the association between PICK1 and insulin granules, we turned to 3D-resolved structured illumination microscopy (SIM). We transduced INS-1E cells with KD + WT and immunostained for eGFP-PICK1 (eGFP antibody) and insulin and assessed the spatial distribution (Figure 8B). Again, we observed overlapping structures between PICK1 and insulin throughout the cell, although this occurred most prominently in the perinuclear region. Moreover, when zooming in on individual granules, we observed numerous structures with either partial overlap of the signal or side-by-side localization, consistent with transient structures in a fission process (Figure 8B).

### 3D-dSTORM enables quantification of PICK1 budding from insulin granules.

**Figure 9:**
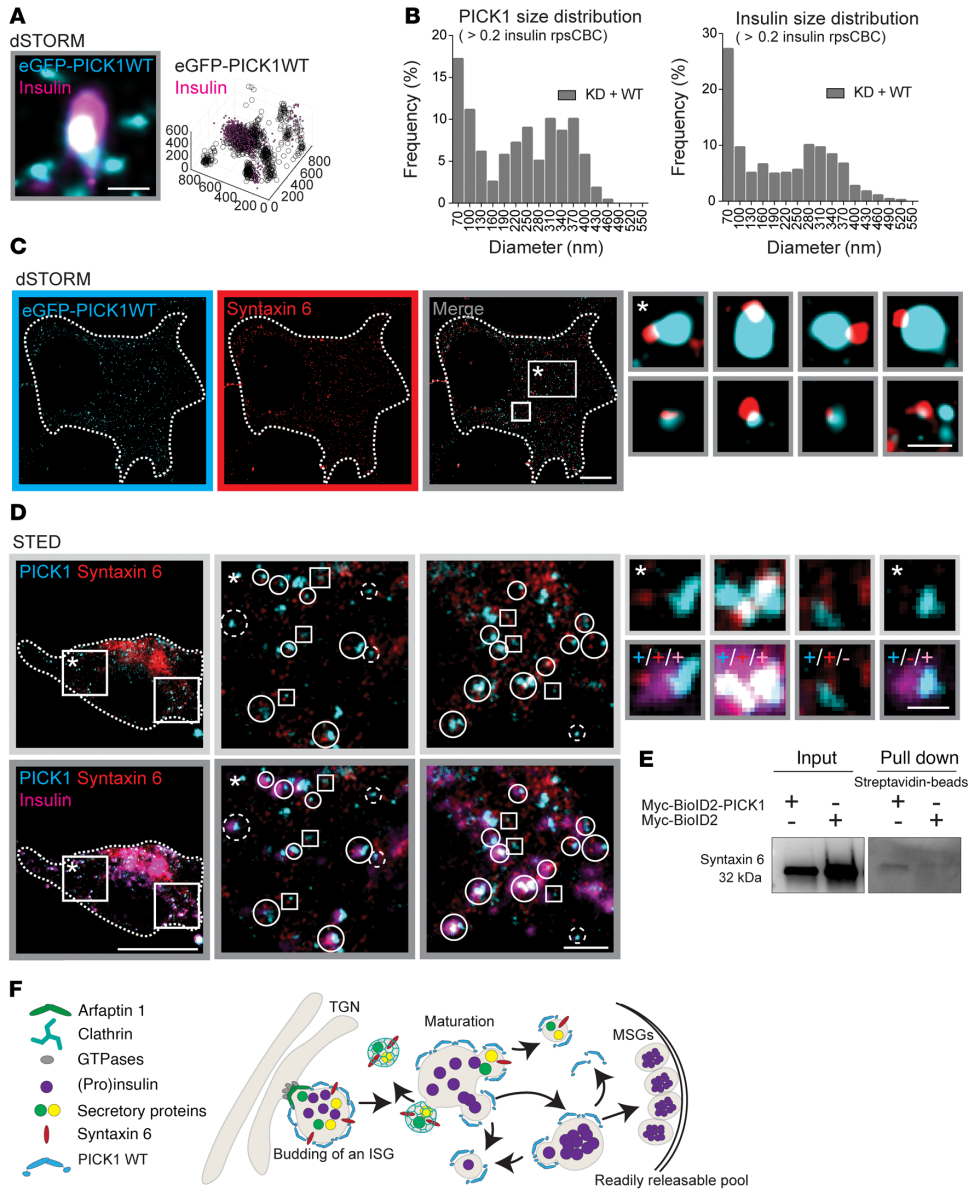
Super-resolution imaging implicates PICK1 in an egress route from ISGs. (**A** and **C**) Representative dSTORM images of INS-1E cells transduced with KD + WT and immunostained for eGFP-PICK1 (cyan), insulin (magenta), or syntaxin 6 (red). (**A**) dSTORM image demonstrates eGFP-PICK1 in small clusters around insulin granules; the 3D illustration shows amounts of insulin (magenta) in the small surrounding structure of PICK1 (black). Axis units indicate nm. Scale bar: 250 nm. (**B**) The size distribution of PICK1 and insulin clusters confirms a high prevalence of small clusters less than 100 nm in the colocalized structures defined as CBC >0.2 insulin in the rpsCBC distribution. (**C**) Representative dSTORM image of INS-1E cells. Scale bar: 5 μm. Right: Inset with higher-magnification image of overlapping PICK1- and syntaxin 6–positive structures. Scale bar: 250 nm. (**D**) Representative STED image of INS-1E cells immunostained for PICK1 (cyan), syntaxin 6 (red), and insulin (magenta). Scale bar: 5 μm. Right panels: Insets at higher magnification. Squares represent colocalized PICK1/syntaxin 6 clusters; dashed circles, colocalized PICK1/insulin clusters; whole circles, colocalized PICK1/syntaxin 6/insulin clusters. Scale bar: 1 μm. Top right: Higher-magnification examples of the different colocalized proteins. Scale bar: 250 nm. (**E**) INS-1E cells were transiently transfected with a Myc-BioID2–PICK1 construct or Myc-BioID2 as control. Biotinylated proteins were pulled down from cell lysates with streptavidin beads and analyzed by immunoblotting for syntaxin 6. *n =* 3 individual experiments. (**F**) Putative model for the role of the IPA N-BAR domain proteins in insulin granule biogenesis. Arfaptin 1 controls the neck of growing ISGs at the TGN in a complex with effector and kinases, while PICK1 — in either a homo- or heterodimeric complex with ICA69 — localizes around the growing ISGs, promoting membrane fission. After the ISGs are budded off, they undergo a maturation process. We propose that PICK1, during multiple budding events, removes excess membrane and cargo from the insulin granules in a process complementary to clathrin-dependent egress.

To increase resolution and enable better visualization as well as quantification of the localization of PICK1 in relation to insulin granules and thereby the putative budding process, we turned to dual-color 3D-dSTORM. We transduced INS-1E cells with KD + WT and immunostained for eGFP-PICK1 and insulin (Figure 8C). We used the insulin signal to define the size of the insulin granules and the eGFP signal to identify PICK1-positive clusters (described in Methods and ref. 24). We observed many examples of full overlap between PICK1 and insulin localizations, as described previously; however, we also observed insulin clusters with all PICK1 localizations skewed to one side, as well as insulin clusters not colocalized with but adjacent to one or multiple clusters of PICK1 of a minimum of 50 nm in size (Figure 8, C and F, and Figure 9A).

To quantitatively describe the degree of overlap between insulin granules and PICK1 clusters, we next took advantage of coordinate-based colocalization (CBC) analysis, with a value of 1 indicating a perfect overlap and –1 representing no overlap. To address which range of CBC values reflected a biologically relevant overlap as opposed to random proximity, we shifted the 2 channels with respect to each other in steps of 100 nm (from 100 to 700 nm) and subtracted the resulting CBC histograms to derive random proximity subtracted CBC (rpsCBC) histograms (Figure 8, D and E, and Supplemental Figure 9). Shifts beyond 500 nm did not change the CBC histograms further, and consequently all rpsCBC histograms were derived as original CBC histogram subtracted by the 500 nm–shifted histograms. Importantly, high CBC values (>0.5) were significantly enriched in the rpsCBC histogram relative to the histogram with all CBC values, indicating that these values were nonrandom, whereas values below CBC = 0 were depleted in accordance with their predicted random nature. The full rpsCBC histogram for PICK1 WT ranged continuously from 0.9, indicating almost full overlap, down to 0, indicating weak proximity, with a minor local maximum at 0.8 (Figure 8E). We used 3D-dSTORM to visualize the structures, and Figure 8F shows representative images of colocalized PICK1 and insulin clusters corresponding to different CBC values, ranging from 0.78, reflecting a complete overlap; to 0.61, reflecting one-sided assembly of PICK1; to 0.41, reflecting partially dissociation of PICK1 from the insulin granule; and finally 0.20, reflecting the smaller PICK1 clusters surrounding the insulin granule (Figure 8F). Tentative arrangement of such combined insulin/PICK1 structures according to CBC values conveyed the impression of structures that are coated by PICK1 and bud off insulin granules, which is in accordance with our live confocal microscopy results (Figure 8A, Supplemental Figure 8, and Supplemental Video 1) and SIM images (Figure 8B).

### Super-resolution imaging implicates PICK1 in a novel egress route from ISGs.

We next investigated the PICK1 clusters adjacent to insulin granules, which were mostly less than 100 nm in diameter. In many cases, visual inspection revealed multiple budding processes originating from the same ISG, and in several cases these structures also showed a low number of insulin localizations (Figure 9A). To obtain data to support that these structures were not randomly distributed in proximity to ISG, we extracted the size distribution of PICK1 (Figure 9B) and insulin clusters (Figure 9C) from the section of the rpsCBC histogram (Figure 8E) with values greater than 0.2, which amounted to 9.2% of all the insulin granules (1191 of 12,994) for PICK1 WT. Indeed, both PICK1 and insulin structures displayed bimodal distribution, with a significant fraction of small structures (>100 nm), consistent with a budding process involving PICK1 and insulin.

We next considered the possibility that these PICK1-positive buds on insulin granules represent precursors of vesicles responsible for the removal of excess membrane and generic membrane trafficking proteins, such as syntaxin 6, during the maturation process (13, 37). We transduced INS-1E cells with our lentiviral construct enabling molecular replacement of endogenous PICK1 with eGFP-PICK1 (KD + WT) and immunostained for eGFP-PICK1 and syntaxin 6; we again used dSTORM to evaluate the putative overlay. Indeed, we observed colocalization of small (<100 nm) PICK1 and syntaxin 6 clusters (Figure 9C and Supplemental Figure 10A), consistent with the hypothesis that the 2 proteins bud off from ISGs. However, we also frequently observed small syntaxin 6 clusters on one side of larger PICK1 clusters (~150–200 nm; Figure 9C), resembling syntaxin 6–positive budding processes devoid of PICK1. Consistent with this finding, we also observed small (<100 nm) syntaxin 6 clusters without PICK1 (data not shown).

To verify that these structures were associated with insulin granules, we turned to stimulated emission deletion (STED) microscopy. This enabled us to perform 2-color super-resolution (staining for PICK1 and syntaxin 6) together with confocal microscopy (insulin). We indeed observed numerous insulin granules positive for both PICK1 and syntaxin 6 (Figure 9D); in some cases, PICK1 and syntaxin 6 signals overlapped, and in others they were clearly separated. We also observed several insulin granules with PICK1 but no detectable syntaxin 6 (Figure 9D). Finally, SIM revealed association of clathrin with a subset of PICK1-positive structures, but only with partial overlap (Supplemental Figure 10C).

To biochemically probe the proximity of PICK1 and syntaxin 6, we took advantage of the proximity-dependent biotin identification (Bio-ID2) approach, which uses BirA to biotinylate proteins in close proximity to a bait protein (38, 39). PICK1 was fused with a Myc-BioID2 construct, as bait, and INS-1E cells were transiently transfected with the Myc-BioID2–PICK1 construct. Immunoblotting confirmed the presence of syntaxin 6 in a streptavidin pull-down (Figure 9E), suggesting that PICK1 and syntaxin 6 are indeed in close spatial proximity in INS-1E cells. Similar blotting for clathrin did not show a difference in pull-down between cells transfected with Myc-BioID2 and Myc-BioID2–PICK1 (Supplemental Figure 10D).

In summary, we propose that PICK1 plays a role in egress of vesicles from ISGs carrying small amounts of insulin and generic membrane trafficking proteins. This process appears complementary to clathrin-dependent egress, which is responsible for removing excess membrane and generic membrane trafficking proteins during the maturation process of ISGs (Figure 9F).

### The coding variants in the PICK1 BAR domain increase fission from insulin granules.

**Figure 10:**
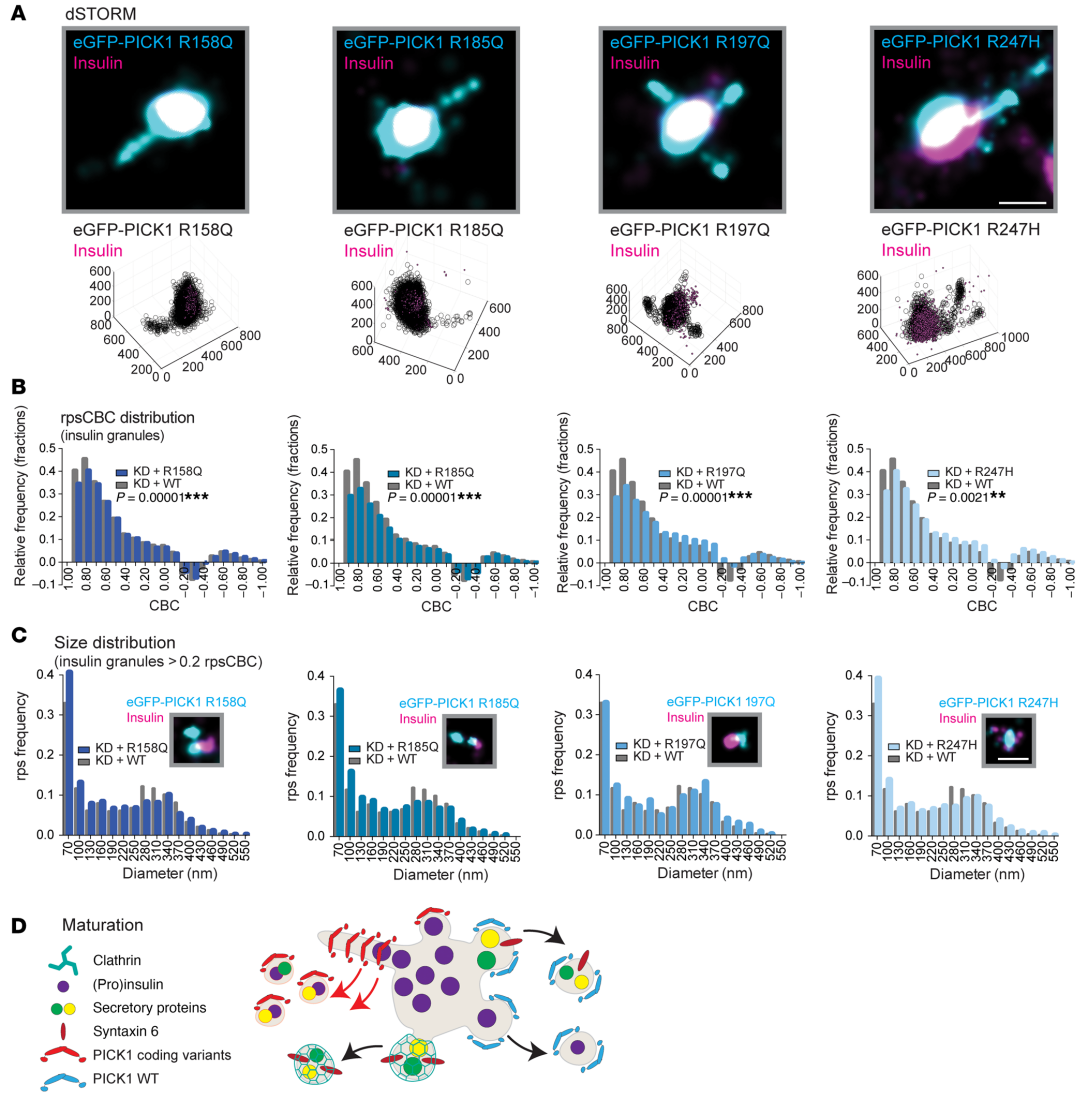
The PICK1 coding variants alter fission from insulin granules. INS-1E cells were transduced with the coding variants and immunostained for eGFP-PICK (cyan) and insulin (magenta). (**A**) Representative dSTORM images of eGFP-PICK1 in tubular structures colocalized with insulin. Scale bar: 250 nm. Bottom row: The same data illustrated with 3D; axis values indicate nm. (**B**) rpsCBC distribution of PICK1 clusters to insulin granules of the 4 coding variants (shades of blue) compared with PICK1 WT (gray). Kolmogorov-Smirnov test was used to test the cumulative distribution, **P < 0.01, ***P < 0.001; *n =* 4–5 individual experiments. (**C**) Size distribution of colocalized insulin granules and PICK1 clusters, defined as rpsCBC >0.2, shown with representative dSTORM images of small insulin clusters colocalized with eGFP-PICK1. Scale bar: 250 nm. (**D**) Proposed model for the PICK1 coding variants in insulin granule biogenesis. We propose that the PICK1 coding variants, with increased abscission efficacy, may cause tubulation and premature budding from the SGs during and/or after the maturation process, generating small clusters that contain excess membrane cargo and insulin.

Next, we expressed each of the coding variants fused to eGFP and inspected 3D-dSTORM images of the transduced INS-1E cells. Similar to PICK1 WT, the coding variants localized to insulin granules (consistent with the confocal data in Supplemental Figure 4, A and B) and also showed PICK1 clusters adjacent to and surrounding the insulin clusters (Figure 10A). Notably, the coding variants were more prone to produce small structures and tubular extensions from the granules than was PICK1 WT (Supplemental Figure 11, A–E), and these structures occasionally also contained insulin (Figure 10A and Supplemental Figure 11, B–E).

We quantitatively assessed whether the coding variants changed the association between PICK1 and insulin granules by comparing the rpsCBC histograms for insulin granules in INS-1E cells expressing the PICK1 coding variants with those expressing PICK1 WT. The rpsCBC histograms for all 4 coding variants differed significantly from the PICK1 WT rpsCBC histogram and showed a reduction in the prevalence of the insulin granules with the highest CBC values (i.e., 0.7–0.9) in comparison to PICK1 WT (Figure 10B). We observed an increase, although less pronounced, in insulin frequency for CBC values ranging from 0 to approximately 0.4 in the R197Q and R247H variants. These data suggest a change in the dynamic association of PICK1 with insulin granules, with the steady state shifted toward lower overlap, which in turn might reflect increased fission or abscission of vesicles from insulin granules.

To further address whether the PICK1 coding variants might affect fission of insulin granules, we examined the size distribution of colocalized insulin granules at CBC values >2 (Figure 10C). Indeed, we observed an increase in frequency of the smallest (≤100 nm) PICK1-associated insulin structures for the R158Q, R185Q, and R247H coding variants compared with PICK1 WT. These results are in agreement with our in vitro studies and indicate that the PICK1 coding variants, surprisingly, might increase the rate of vesicle budding from the insulin granules, consequently generating more small insulin-positive clusters (Figure 10, B–D).

Finally, to address how general this phenotype might be, we mimicked the most prominent of the coding variants (R247H) in *Drosophila* PICK1 (dPICK1 K249H-HA). Immunolabeling of dPICK1 WT-HA in large peptidergic cells in the ventral nerve cord of pupal flies showed localization overlapping with and bordering GFP-Golgin245, suggesting localization at and proximal to the Golgi compartment (Supplemental Figure 12). Strikingly, the dPICK1 K249H-HA construct clearly dissociated from GFP-Golgin245 in bright spots that in some case showed distinct tubular shapes (Supplemental Figure 12), suggesting that the R247H coding variant interfered with a structural and functional feature of the PICK1 BAR domain that is preserved across species and may affect the early RSP in many (neuro)endocrine cell types.

## Discussion

The IPA group of N-BAR domain proteins has recently been implicated in insulin granule biogenesis, presumably regulating and inducing membrane deformation of secretory granules (SGs) both during fission of the nascent TGN membrane, leading to generation of ISGs, and during the maturation process, leading to formation of MSGs (19–22). Here, we report 4 coding variants in the PICK1 BAR domain that were identified in a WES of exomes of Danish patients with T2DM. All 4 missense mutations displayed compromised function in relation to SG biogenesis in insulin-producing cells, with 2 of the mutations also showing a functional dominant-negative effect on the WT protein. In line with the membrane-binding properties of the BAR domain, the coding variants caused subtle changes in the interaction of PICK1 with liposomes. For 2 of the variants, liposome binding strength and cooperativity were reduced, but surprisingly membrane fission/abscission efficacy was increased. This prompted us to probe the dynamics of the association of PICK1 with ISGs by use of both live confocal microscopy and super-resolution microscopy. Our data demonstrated that association with insulin granules is transient, underscored by PICK1’s steady-state association with approximately 10% of insulin granules. Further, our data supported a role for PICK1 in an egress process from ISGs through vesicular budding events that assists in or complements clathrin-dependent removal of excess membrane and generic membrane trafficking proteins. Finally, quantitative colocalization analysis of super-resolution data suggested that the steady-state distribution of the PICK1 coding variants shifted away from full coverage of insulin granules and toward budding structures and small granules, consistent with increased fission/abscission efficacy.

It is clear that PICK1 dysfunction is not a major determinant in the development of T2DM. However, although only 5 of the 1000 T2DM patients in the study had coding variants in the PICK1 gene, disruptive mutations may in rare cases contribute to the disease. The within-study odds ratio was 5, but it must be emphasized that the power of the study was too low (*P =* 0.22, Fisher’s exact test) to conclude a significant association between T2DM and BAR domain coding variants, even if they phenotypically are considered as one. The same is true for the 2 dominant-negative variants, R158Q and R247H, considered together (*P =* 0.12, Fisher’s exact test); however, both of these variants are rare SNPs, with a frequency of A = 0.00003 for R158Q and A = 0.00002 (GnomAD_exome and NHLBI Trans-Omics for Precision Medicine [TOPMed]) for R247H, suggesting that the likelihood for random hits in our sample of 1000 patient was very low. Higher-powered studies are needed to determine whether coding variants in the PICK1 BAR domain are indeed part of the complex genetic makeup predisposing to T2DM or possible other PICK1-related pathological conditions.

The causal relation to disease aside, the finding of compromised function of the coding variants in supporting insulin storage and dominant-negative function even in primary β cells provided important insight into the molecular function of BAR domain proteins and in particular the functional role of PICK1. Relatively few disease-related mutations in BAR domains have been characterized previously (see, e.g., refs. 40, 41), and consequently, the current understanding of BAR domain structure function reflects hypothesis-driven mutations of positively charged residues, which show reduced liposome binding and altered cellular localization (16, 28, 42). Consistent with these findings, the mutated arginines in our study compromised clustering of PICK1 in COS-7 cells; however, their overall localization to the RSP in INS-1E cells was intact. Interestingly, our TEM studies demonstrated that membrane deformation by PICK1 WT primarily resulted in relatively large adherent liposomes, suggesting the propensity of PICK1 to elicit abscission-like scission of liposomes rather than tubulation as seen for other N-BAR proteins. This is in accordance with the punctate rather than tubular localization pattern of PICK1 in cells even upon overexpression.

Previous coarse-grained molecular dynamics simulations of the endophilin N-BAR domain on liposomes evolving to tubular networks (reticulation) showed early stages of deformation, termed budding, with endophilin lining up at the bottom of furrows to bulge out membrane buds an order of magnitude larger than would fit the curvature of the BAR domain (43). Notably, our recent small-angle X-ray scattering structure of PICK1 demonstrated elongated oligomers of PICK1 (44). Here we propose that this linear arrangement of PICK1 might evolve such initial buds into abscission events rather than a tubular network.

On the other hand, TEM studies of liposome deformation by R197Q or R247H revealed tubular structures, while liposomes incubated with R158Q, R185Q, or R247H formed high-curvature nascent buds and small vesicular structures. Fission efficacy for N-BAR proteins was previously described as a balance between the fission-promoting capacity of amphipathic helices and the fission-restraining action of the oligomerized BAR domain, which stabilize tubular structures (17), although this view has been contested (34, 43). For PICK1, we propose that the abscission-like fission of insulin granules, which is recapitulated in the purified system and observed by electron microscopy, results from a fine-tuning of the counteracting forces of the amphipathic helix and the BAR domain. Such competing mechanisms might also explain the low Hill coefficient (negative cooperativity) of the dose dependence of fission. For the coding variant, in which the BAR domain is compromised, the fission capacity of the helix becomes relatively more dominant, leading to higher-curvature membrane structures and a dose dependence with Hill coefficients greater than 1. Notably, such increased abscission efficacy provides a credible mechanistic explanation for the dominant-negative function of R158Q and R247H. Also, we note that R197Q, which was identified in both a patient and a healthy participant, functionally appeared to diverge the least from PICK1 WT. Although this variant clustered less than PICK1 WT in COS-7 cells, associated less prominently with insulin granules, and failed to substitute for PICK1 in rescuing insulin storage in INS-1E cells, it retained the abscission-like scission capacity and low Hill coefficient in the biophysical experiments and also did not increase the fraction of small insulin granules in INS-1E cells, presumably explaining its lack of dominant-negative function. Inspection of our AlphaFold2 model (Supplemental Figure 1) suggested that the positively charged residues were all involved in charge-charge interaction with negatively charged residues to stabilize helix-helix interactions within (R158, R185, and R247) or between the BAR domain monomers. Remarkably, the 2 dominant-negative variants were located the closest to the concave surface, thereby presumably affecting membrane association the most.

A comparable set of disease-associated mutations in the BAR domain protein bridging integrator 1 (BIN1) — D151N and R154Q, located slightly closer to the tip of the BAR domain — have previously been shown to also affect BAR domain oligomerization (40). Similar to the PICK1 coding variants, the BIN1 mutations reduced membrane association without affecting curvature sensing. However, unlike the coding variants of PICK1 in our study, they compromised tubulation as a result of reduced oligomerization, highlighting that subtle perturbations to the oligomerization interface may dramatically alter the ability of N-BARs to shape lipid membranes. Ultimately, further insight into the membrane-molding action of PICK1 and the changes imposed by the coding variants, e.g., in relation to oligomerization of PICK1, will rely on high-resolution structural data on lipid membranes.

The ability of PICK1 to deform lipid membranes in a cellular context was originally associated with budding of SGs from the TGN in growth hormone– and insulin-secreting cells (20, 21). Surprisingly, we did not see changes in proinsulin levels upon PICK1 KD, as reported previously (20, 22), or after reexpression of the coding variants, suggesting that the function of PICK1 in INS-1E cells is likely more prominent after budding from the TGN. The subsequent process of SG maturation entails removal of excess material such as syntaxin 6, vesicle-associated membrane protein 4 (VAMP4), furin, and both mannose 6–phosphate receptors (MPRs) from the SGs. This process relies on clathrin as the coat protein, as well as AP-1A and Golgi-localized, γ-ear–containing, ADP-ribosylation factor–binding proteins (GGAs) as adaptors recruited by ARF1 (12, 14, 45). Several lines of evidence presented here imply that the function of PICK1 extends to not only the initial budding from TGN, but also the egress from ISGs (see Figures 8 and 9). Consistent with this idea, we recently demonstrated that molecular replacement of PICK1 with a fission-incompetent PICK1 variant with mutations in the amphipathic helix in the N-BAR domain region (PICK1 V121E–L125E) resulted in larger insulin granules (24). Yet the combined in vitro experiments and cellular imaging suggested that the functional effect of the coding variants identified in patients with T2DM actually accelerated PICK1 egress from ISGs (see Figure 10D). It is unclear how this may compromise insulin storage, but the tendency to produce more, small (≤70 nm) PICK1-associated insulin granules suggests that insulin may to some extent follow egress if the process takes place prematurely, e.g., prior to the pH- and Ca^2+^-dependent condensation of insulin (see Figure 9D).

In summary, 4 coding variants in the PICK1 BAR domain discovered by WES in a group of Danish patients with T2DM revealed genotype-phenotype relations that are relevant for BAR domain proteins in general. Further, these mutations pointed us to a role for PICK1 in a what we believe to be a novel trafficking pathway involved in maturation of insulin granules. We propose that this pathway might serve to regulate removal of insulin for, e.g., lysosomal or autophagocytic degradation in response to changes in glucose concentration, but this requires further study.

## Methods

### Cell cultures

COS-7 cells, a cell line derived from an African green monkey fibroblast, were cultured in were cultured in DMEM 1885 (Substrate and Sterile Central [SSC], University of Copenhagen) containing 10% FBS, 1% penicillin/streptomycin (P/S), and 1% l-glutamine at 37°C in a humidified 10% CO_2_ atmosphere. INS-1E cells, an insulin-producing cell line derived from a rat β cell, were maintained in RPMI 1640 (with HEPES and 1% l-glutamine) or RPMI 1640 (with HEPES + GlutaMAX; SSC, University of Copenhagen) medium, both containing 10% FBS, 1% P/S, and 1.5% 100× RPMI supplement (1 mM Na pyruvate and 50 μM 2-mercaptoethanol) at 37°C in a humidified 5% CO_2_ atmosphere. The GRINCH cells are insulin-producing cells with stable expression of hPro-CpepsfGFP (gift from Peter Arvan, University of Michigan Medical School, Ann Arbor, Michigan, USA) (36). GRINCH cells were maintained in RPMI 1640 (with HEPES) medium including 10% heat-inactivated FBS, 1% P/S, 1 mM Na pyruvate, 2 mM l-glutamine, and 50 μM 2-mercaptoethanol at 37°C in a humidified 5% CO_2_ atmosphere.

### Molecular biology — DNA constructs

The pET41 vector encoding GST-PICK1 for bacterial expression (46) and the peYFP C1 vector encoding YFP-PICK1 (29) have been described previously. Both vectors were edited using the QuikChange kit (Stratagene) to generate constructs encoding PICK1 coding variants: R158Q, R185Q, R197Q, and R247H.

The FUGW vectors encoding shRNA targeting rPICK1 with expression of eGFP (KD) and eGFP-rPICK1 (KD + WT) were gifts from Robert C. Malenka, Stanford University, Stanford, California, USA (31). The shRNA targeting rPICK1 was deleted to generate the ctrl vector as described previously (24). The PICK1 coding variants were introduced into the KD + WT FUGW vector using the QuikChange kit. The pHSynXW vector encoding eGFP-rPICK1 was a gift from Richard Huganir, Johns Hopkins University, Baltimore, Maryland, USA. We replaced eGFP with mCherry using the QuikChange kit. The Myc-BioID2 construct was fused with PICK1 by a linker region (GGGS), generating myc-BioID2–PICK1, purchased from Thermo Fisher Scientific. Myc-BioID2 was removed from PICK1 and used as a ctrl.

### Lentivirus production

Lentivirus production was carried out as described previously (24). In short, HEK293T cells were cotransfected with the appropriate FUGW vectors and 2 helper plasmids (pBR8.91 and pMDG [PlasmidFactory]) using Lipofectamine (Invitrogen, Life Technologies) and 10 mM Na butyrate. The supernatant was collected and ultracentrifuged at 72,000*g* for 2 hours. The pellet was resuspended in MEM medium (Gibco, Thermo Fisher Scientific) and stored at –80°C.

### Protein expression and purification

*E. coli* (BL21 DE3 pLysS) cultures were transformed with pET41 plasmids encoding either GST-PICK1 WT or the 4 PICK1 coding variants. Protein expression and purification were performed as described in Supplemental Methods.

### Liposome preparation

We prepared unilamellar liposomes from bovine brain extract (Type I, Folch fraction I, Sigma-Aldrich) by following a standard hydration/extrusion procedure (18), which is further described in Supplemental Methods.

### Flow cytometry

We modified the previously described SLIC assay (47) to a high-throughput assay by use of a FACS Fortessa (5 laser; BD Biosciences). See Supplemental Methods for the procedure.

### TEM imaging

Purified PICK1 WT and PICK1 coding variants were preincubated with liposomes for 1 hour at room temperature, with final concentrations of 0.3 μM protein and 0.001 μg/mL liposomes. Grids were prepared by glow discharging for 30 seconds. Protein-liposome mixture (5 μL) was added to the grids and incubated for 1 minute before 5 μL 2% uranyl acetate was added for an additional minute of incubation. The grids were washed with sterile H_2_O, and filter paper was used to remove excess liquid. The grids were examined with a Philips CM 100 TEM at 80 kV, and the images were acquired with an Olympus Veleta CCD camera.

### Transient transfection and transduction

One day prior to transfection, cells were seeded out on polyornithine-coated coverslips in 6-well tissue culture plates (TPP, Sigma-Aldrich). Transfection was performed using Lipofectamine (Invitrogen, Thermo Fisher Scientific) and optiMEM (Gibco, Thermo Fisher Scientific), following the manufacturers’ protocol, with a ratio of Lipofectamine to DNA of 3:1. 0.5 and 1 μg DNA/mL was used for COS-7 and INS-1E cells, respectively. Transfection was set to 5 hours or overnight, after which optiMEM was replaced by culture medium. Experiments were carried out 48 hours after transfection. For live-cell microscopy, cells were seeded on polyornithine-coated Lab-Tek II 8-well chambers (Nunc, Thermo Fisher Scientific) at a density of 20,000 cells/well or on poly-l-lysine–coated MatTek microwell dishes at a density of 60,000 cells/dish.

To optimize transduction efficiency, transduction was performed by spinoculation. 20 μL of the lentiviral suspension was added to 3 × 10^6^ INS-1E cells in 3 mL reheated medium and centrifuged at 800*g*, 32°C, for 2 hours. The supernatant was aspirated, and INS-1E cells were resuspended in preheated culture medium and transferred to 75T tissue culture flasks (TPP, Sigma-Aldrich). INS-1E cells were incubated for a minimum of 4 days to recover and initiate expression.

### Pancreatic islet isolation and dissociation into single cells

Pancreatic islets from 12 week-old male C57BL/6NRj mice (Janvier Labs) were isolated by bile duct perfusion using liberase as described in Supplemental Methods. Four days after isolation, islets were washed in HBSS and dissociated into single cells by gently pipetting up and down for 3 minutes in the presence of 0.2% trypsin in HBSS. Trypsinization was stopped in 2% human serum (HS) RPMI, cells were centrifuged at 200*g* for 4 minutes, and the pellet was resuspended in 2% HS RPMI. Approximately 400–500 islets were pooled from 2–3 mice per experiment. The dispersed β cells were seeded on laminin-coated Lab-Tek Permanox 4-well chambers (Nunc Merck) in 2% HS RPMI and incubated overnight to recover before transduction. 5 μL of the lentiviral suspension was mixed with 400 μL reheated 2% HS RPMI per well and added to the β cells for 6 hours before substitution with new culture medium. The β cells were incubated for 6 days to recover and initiate expression. For every experiment, 1 well was used as a ctrl with no transduction.

### Immunocytochemistry

#### Antibodies.

Antibodies used for immunostaining and immunoblotting are described in detail in Supplemental Methods. See complete unedited blots in the supplemental material.

#### Immunostaining.

Cells were seeded on polyornithine-coated coverslips in 6-well tissue culture plates with a density of 250,000 cells/well (COS-7 cells) or 500,000 cells/well (INS-1E cells) 4 days prior to fixation. Staining of INS-1E cells for dSTORM imaging was prepared as described previously (24); for the general staining procedure, see Supplemental Methods.

#### Bio-ID.

Magnetic streptavidin-coupled Dynabeads (Thermo Fisher Scientific) were used to isolated biotinylated proteins from transduced INS-1E cells. See Supplemental Methods for the procedure.

#### Light microscopy.

Confocal microscopy was performed on fixed coverslips using an inverted laser-scanning microscope (LSM) 510 or an upright 710 or 900 (Zeiss), all with 63×/1.4 numerical aperture (NA) oil immersion Apochromat objectives. Confocal imaging of INS-1E cells was primarily performed in a blinded manner. For settings, see Supplemental Methods.

Live-cell images of GRINCH cells were acquired on an inverted Nikon A1+ point scanning confocal microscope (Nikon) with 405, 488, 561, and 647 nm lasers and a 60×/1.4 NA oil immersion Apochromat objective. Cells were kept in Krebs-Ringer solution (20 mM HEPES [pH 7.4], 119 mM NaCl, 4.75 mM KCl, 2.54 mM CaCl_2_, 1.2 mM MgSO_4_, 1.18 mM KH_2_PO_4_, 5 mM NaHCO_3_) supplemented with 0.2% BSA and 11 mM glucose in a heated and humidified chamber. *Z*-stack live imaging was performed with a total coverage of 0.500 μm in the *z* plane. The duration of recordings was 2–4 minutes per session. The GFP and mCherry fluorescent signals were detected using the 488 and 568 nm lasers, respectively.

#### Super-resolution microscopy.

dSTORM images were acquired on an ECLIPSE Ti-E epifluorescence/TIRF microscope (Nikon) with 405, 488, 561 and 647nm lasers (Coherent) and a 100×/1.49 NA oil immersion Apochromat objective. Settings and conditions for dSTORM imaging are as previously described (24).

dSTORM imaging on fixed coverslips was performed in a buffer containing β-mercaptoethanol and an enzymatic oxygen scavenger system (10% [w/v] glucose, 1% [v/v] β-mercaptoethanol, 50 mM Tris-HCl [pH 8], 10 mM NaCl, 34 μg/mL catalase, 28 μg/mL glucose oxidase). For settings, see Supplemental Methods.

Airyscan 2 images were obtained on fixed coverslips using an inverted LSM 980 (Zeiss) with a 63×/1.4 NA oil immersion Apochromat objective and a Airyscan 2 detector (a 32-channel GaAsP photomultiplier tube area detector). DAPI, Alexa Fluor 488, Alexa Fluor 568, and Alexa Fluor 647 fluorescent signals were detected using diode lasers at 405, 488, 561, and 639 nm, respectively.

SIM was performed on room-temperature-fixed coverslips using an Elyra PS.1 microscope (Zeiss) with 488, 561, and 642 nm diode lasers (Coherent) and a 63×/1.4 NA oil immersion Apochromat objective. *Z*-stack images were acquired from the top to bottom of cells (0.110 μm per interval), and channels were imaged separately.

STED images were acquired on fixed coverslips using a STEDYCON with 488, 561, and 640 nm lasers together with a 775 nm STED laser (all pulsed) (Abberior Instruments GmbH). The STEDYCON was attached to a Zeiss Axio imager Z2 with a 100×/1.46 NA oil immersion Apochromat objective. The 488 nm laser was used to obtain confocal resolution in cells stained for guinea pig anti-insulin and Alexa Fluor 488–conjugated goat anti–guinea pig antibody.

Analysis of super-resolution images is described in Supplemental Methods.

#### Confocal image analysis.

Image processing was primarily performed using ImageJ (NIH). Cells in confocal images were outlined by region of interest (ROI) in the 488 nm channel (YFP/eGFP) in combination with the PICK1 channel to identify transfected/transduced cells. Prior to quantification of the immunosignal, background noise was subtracted, and a threshold was set for each channel. These settings were held constant throughout each individual experiment. The images were converted to binary images and multiplied into each ROI of the original image, which gave a total intensity value. To analyze the clusters, an “analyze particles” filter (available in ImageJ) was added with a minimum of 5 pixels per cluster and with a circularity of 0.10 to 1.00 before multiplying the binary image with the original image. For total immunosignal, immunosignals from the transfected/transduced cells within each image were normalized to those from the untransfected/untransduced cells within the same image. Quantification was performed in a blinded manner. Plotting of PICK1 and insulin immunosignals per cell, shown with linear regression, excluded levels above those for the ctrl cells for each individual experiment.

Analysis of PICK1 clusters in COS-7 cells was performed using Igor Pro version 6.34A software (WaveMertrics). Clusters were separated by ROIs and chosen based on the following conditions: a minimum area of 5 pixels, maximum deviation of 75%, fluctuation factor of 1, and minimum ellipticity of 0.5. Background threshold was determined for each image due to high variation in cluster intensity. For presentation, the images are shown in inverted gray scale.

### Statistics

Data were transferred to GraphPad Prism software (GraphPad Software) for statistical analysis and presentation. Data were tested for normality using either the D’Agostino-Pearson normality test or the Shapiro-Wilk normality test. For data with normality, we used 1-way ANOVA followed by either Dunnett’s or Turkey’s multiple-comparison test for statistical comparison, while data with no normal distribution were tested for statistical comparison using the nonparametric Kruskal-Wallis test followed by Dunn’s multiple-comparison test. Statistical significance for cumulative distribution was compared using the Kolmogorov-Smirnov test in MATLAB (MathWorks). Data are presented as mean ± SEM. *P* values less than 0.05 were considered significant. *n* represents the number of cells or individual experiments performed. The built-in GraphPad analysis “identify outliers” was used for the data set in Figures 3, 4, and 6.

## Author contributions

In vitro work was conducted by RCA, JHS, JR, NRC, MS, GNH, and TTEN. Cellular experiments were performed by RCA with help from DSS, JR, AMJ, and MP. β Cell work was conducted by RCA, SSP, and MAO. Microscopy imaging was acquired by RCA, JHS, and JR with help from with help from AMJ. VKL and RCA conducted the *Drosophila* experiments. RCA, MDL, JHS, JR, NRC, DSS, VKL, AMJ, JBS, OK, ADA, KLM, and UG designed the experiments. Data analysis and interpretation of the results were performed by RCA, MDL, JHS, JR, NRC, VKL, TTEN, MPK, RH, OK, ADA, BH, UG, and KLM. The manuscript was written by RCA and KLM.

## Supplementary Material

Supplemental data

Supplemental video 1

## Version 1 01/25/2022

In-Press Preview

## Version 2 03/01/2022

Electronic publication
