# Supplementary material for: Coding variants identified in patients with diabetes alter PICK1 BAR domain function in insulin granule biogenesis
Source: J Clin Invest. 2022 Mar 1;132(5):e144904. doi: 10.1172/JCI144904 (PMC8884907; doi:10.1172/JCI144904)

Supplemental methods

Drosophila genetics

The fly lines carrying the UAS-driven *PICK1A*^{WT}*-HA* and UAS-driven *GFP-Golgin245* transgenes (1) and the *PICK1*^{1} and *PICK1*^{2} deletion alleles (2) have been described previously. The *c929-Gal4* driver for expression in large high-capacity peptidergic neurons (3) was a gift from Dr. Paul Taghert, Washington University, Missouri, USA. The coding variant R247H corresponds to K249H in *Drosophila*, and The HA-tagged *PICK1A*^{K249H} transgene was generated in a similar way as UAS-PICK1A^{WT}-HA and inserted into the same attP acceptor site on the 3rd chromosome (M{3xP3-RFP.attP]ZH}-86Fb attP) using Phi31C recombination. In brief, the preexisting pUASTattB-PICK1A-HA construct was mutagenized by megaprimer mutagenesis to introduce mutations into the *PICK1A* coding region corresponding to the K249H alteration of the protein sequence. Embryo injections and transformant selection was performed by BestGene Inc. (CA, USA). Flies were reared on standard cornmeal food at 25°C.

Genotypes used for immunostaining: WT PICK1A-HA rescue (*w*^{1118}*; w+ c929-Gal4 PICK1*^{1} */PICK1*^{2}*; w+ M{3xP3-RFP.attP}ZH-86Fb UAS-PICK1A-HA/w+ UAS-GFP-Golgin245*), PICK1A^{K249H}-HA rescue (*w*^{1118} *; w+ c929-Gal4 PICK1*^{1} */PICK1*^{2} *; w+ M{3xP3-RFP.attP}ZH-86Fb UAS-PICK1A*^{K249H}*-HA/w+ UAS-GFP-Golgin245*).

Immunostaining in *Drosophila* peptidergic somata

To visualize PICK1-HA and GFP-Golgin245 localization in *Drosophila* peptidergic somata, pharate adult stage pupae were dissected on Sylgard slabs in PBS and fixed in 4% formaldehyde for 30 min on ice. Washed 6 x 10 min in PBX (PBS + 0.3% Triton X-100), blocked in 5% GS in PBX for 30 min at RT, and incubated with primary antibody in blocking buffer over night at 4°C. This was followed by 6 x 10 min washes in PBX at

RT and incubation with secondary antibody in PBX with 5% GS for 2 hours at RT. Finally, the samples were washed in 6 x 10 min in PBX, 2 x 5 min in PBS and mounted on glass slides using Prolong® Gold antifade mounting reagent. The intrinsic GFP fluorescence was visualized directly without immunolabelling.

Pancreatic islet isolation

Pancreases from 12 week-old male C57BL/6NRj mice (Janvier, Saint Berthewin Cedex, France) were infused via the common bile duct with liberase solution (0.1 mg/mL Liberase TL (Roche, Denmark), 0.1 mg/mL DNase I, 25 mM $CaCl_2$ in HBSS with Ca^{2+} and Mg^{2+} (Life Technologies)). The pancreases were excised and incubated in liberase solution at 37°C for 17 min after which the tubes were shaken vigorously for 1 min. Digestion was terminated by ice-cold HBSS supplemented with 2.7 mM glucose (Sigma-Aldrich) and 0.3% bovine serum albumin (BSA) (Sigma-Aldrich) and the tubes were centrifuged at 200 g at 4°C for 3 min. After two washes, the islets were separated from exocrine tissue by filtration through a 400 µm wire sieve (Buch & Holm), a 100 µm and 70 µm cell strainer (Merck), respectively. The islets were hand-picked and incubated in RPMI 1640 with *GlutaMAX* (Gibco, Life Technologies) supplemented with 10% FBS (Biosera) and 1% P/S (Gibco) in cell culture dishes (DACOS), at 37°C in a humidified 5% CO_2 atmosphere. One day post isolation, the islets were transferred to RPMI 1640 with 2% human serum (HS) (BioWhittaker).

Antibodies

Primary antibodies for immunostaining: mouse anti-clathrin (BD Biosciences, 610499) (1:500), chicken anti-GFP (Abcam, ab13970) (1:2000), rat anti-HA (Roche, clone 3F10) (200 pg/ml), rabbit anti-ICA69 #1, (custom-made, described in (4)) (1:400), chicken anti-PICK1 (Novus Biologicals, NBP1-42829) (1:500), mouse anti-PICK1 (custom-made, 2G10 clone, described in (2)) (1:500), rabbit anti-insulin (Cell signaling, C27C9, #3014) (1:1000), guinea pig-anti insulin (Abcam, ab7842) (1:500), rabbit-anti

TGN38 (Sigma-Aldrich, T9826) (1:2000), rabbit anti-syntaxin 6 (Synaptic Systems, 110-062) (1:2000). Secondary antibodies: Alexa Fluor® 488 conjugated goat anti-chicken (Abcam, ab150173) (1:500), Alexa Fluor® 568 conjugated goat anti-mouse/rabbit (Thermo Fisher Scientific, a11031/a11036) (1:500), Alexa Fluor® 647 conjugated goat anti-mouse/rabbit (Thermo Fisher Scientific, a21236/a21245)) (1:500) and Alexa Fluor® 647-conjugated goat anti-rat (Invitrogen, A21247) (1:500). For STED imaging following secondary antibodies were used: Abberior® STAR far red 640 goat anti-rabbit (Abberior GmbH) (1:500), Abberior® STAR orange 561 goat anti-mouse (Abberior GmbH) (1:500) and Alexa Fluor® 488 conjugated goat anti-guinea pig (Abcam, ab150185). The Abberior ® secondary antibodies were kindly provided by Abberior instruments.

Antibodies for dSTORM immunostaining: rabbit anti-insulin (Cell signaling, C27C9, #3014) (1:500), CF® 568 (Biotium) conjugated donkey anti-rabbit (Jackson ImmunoResearch Labs, 711-005-152) and Alexa Fluor® 647 (Invitrogen) primary conjugated chicken anti-GFP (Abcam, ab13970) or GFP nanobody (Chromotek, gt-250).

Antibodies for immunoblotting: chicken anti-GFP (Abcam, ab13970) (1:1000), rabbit anti-ICA69 #1 (custom-made (4)) (1:400), mouse anti-PICK1 (NeuroMab, Q9NRD5 or custom-made, 2G10 clone) (1:500), rabbit anti-chicken-HPR (Invitrogen, 31401) (1:10,000), goat anti-mouse-HRP (Invitrogen, 31430) (1:10,000) and goat anti-rabbit-HRP (Invitrogen, 31460) (1:10,000). Loading control: HRP-conjugated anti-β-actin (Sigma-Aldrich, A3854) (1:20,000).

Antibody conjugation

Secondary antibodies used for dSTORM were fluorescently labelled by NHS-ester fluorophore conjugation. 50 µL antibody solution (donkey anti-rabbit, chicken anti-GFP or GFP nanobody) (1.2mg/mL) was added to 6 µL $NaHCO_3$ (1M) and 1.25 µL NHS-ester fluorophore (CF®568 or Alexa Fluor® 647) (1mg/mL). The mixture was incubated

for 2 hours at RT and antibodies were isolated using illustra™ NAP-5 columns (GE Healthcare Life Sciences™), resulting in an average labeling rate between 0.8 and 1.

Immunostaining

Cells were washed in ice-cold PBS and fixed with 4% paraformaldehyde, 10 min on ice and 10 min at RT. COS7 cells were washed with PBS and milliQ, and mounted on a glass slide with Prolong® Gold antifade mounting reagent. INS-1E cells were washed in PBS and permeabilized for 30 min in PBS containing 0.2% saponin and 5% goat serum (GS). Subsequently, cells were incubated with primary antibodies (diluted in PBS with 5% GS) for 1 h at RT or overnight at 4°C and washed in PBS prior to incubation with secondary antibodies (diluted in PBS with 5% GS) for 30 min. Cells were washed in PBS and milliQ before mounted on a glass slide using Prolong® Gold antifade mounting reagent. Glass slides were stored in the dark at 4°C. β-cells were fixated and immunostained following the same protocol as for INS-1E cells but mounted on a glass slide using DAPI Fluoromount-G® (SouthernBiotech).

Cell lysates

For immunoblotting and ELISA, cells were washed in ice-cold PBS prior to lysis in 1xRIPA buffer (10 mM Tris-HCl (pH 7.4), 150 mM NaCl, 1% Triton X-100, 1 mM EDTA, 1mM EGTA, 0.5% NP-40) containing 1 mM PMSF or cOmplete protease inhibitor cocktail tablets and phosphatase inhibitor cocktail 3 (Sigma-Aldrich). Cells were scraped off, centrifuged at 11,000 g for 40 min at 4°C and the supernatant was stored at -20°C. The protein content was determined using the bicinchoninic acid assay kit (Pierce™, Thermo Fisher Scientific) to adjust protein concentration for immunoblotting and to calculate relative proinsulin and insulin content.

Immunoblotting

Protein lysates were mixed with 5xsodium dodecyl sulfate polyacrylamide gel electrophoresis (SDS-PAGE) loading buffer, boiled at 95°C for 5 min before loaded into an Any-kD or 4-15% precast gel (Mini-ProTEAN® TGX™, BIO-RAD) and run at 100V for 1-2 hours depending on the protein of interest. The size-separated proteins were transferred to polyvinylidene difluoride membranes (BIO-RAD) for 2 hours at 18 V or for 10 min at 25 V on a trans-blot turbo transfer system (Bio-Rad) and blocked for 1 h in 5% milk in wash buffer (PBS with 0.1% Tween-20). Membranes were incubated with primary antibodies for 1 h at RT or overnight at 4°C, washed 3 times and incubated with HRP-conjugated secondary antibodies for 30 min. Membranes were washed 3 times before developed using either the SuperSignal ELISA Femto Substrate (Thermo Fisher Scientific) or ELC Prime Western Blotting system (Sigma-Aldrich) and captured with a cooled CCD camera.

Co-immunoprecipitation

$15x10^6$ transduced INS-1E cells were seeded out in tissue culture flasks three days prior to lysis. Cell lysis were performed as described above, using lysis buffer (20 mM Tris-HCl (pH 7.4), 150 mM NaCl, 10 mM MgCl and 0.5% NP-40) containing cOmplete protease inhibitor cocktail tablets and phosphatase inhibitor cocktail 3. 50 μg protein lysate per condition was saved for input and 500-1000 μg protein per condition was used for immunoprecipitation analysis. The protocol from the manufacturer was followed. In short, the protein lysate was transferred to prewashed (in lysis buffer) GFP-Trap® magnetic particles (M-270, ChromoTek) and incubated on a rotator for 1 h at 4°C. Particles were separated from the supernatant by using a magnetic DynaMag rack (Invitrogen, Thermo Fisher Scientific). The particles were washed three times in wash buffer (10 mM Tris/Cl (pH 7.4), 150 mM NaCl, 0.05% NP-40, 0.5 mM EDTA) before mixed with 2xSDS-PAGE buffer and boiled for 5 min at 95°C.

Immunoprecipitated protein and input were assessed by immunoblotting. The images were analyzed using the ImageJ software program (Rasband W. S., ImageJ, U.S. National Institutes of Health, Bethesda, MD, USA).

Bio-ID

48 $10x10^6$ INS-1E cells were seeded out in tissue culture flasks a day prior to transient transfection. Transfection was performed as mentioned above, 5 µg DNA (in optiMEM) was used per flask and 5 hours after substituted with F10-DMEM media (Thermo Fisher Scientific). hours post-transfection cells were washed with PBS and scraped off. The cell suspension was centrifuged at 1000 g for 5 min at 4°C, supernatant was aspirated and the pellet was snap frozen using liquid nitrogen. The pellet was resuspended in lysis buffer (50 mM Tris-HCl, 150 mM NaCl, 1% Triton X-100, 0.1% SDS, 0.5% sodium deoxycholate, pH 7.4), rotated for 1 h at 4°C and centrifuged at 13,000 g for 15 min at 4°C. Subsequently the supernatant was transferred to prewashed (in lysis buffer) sepharose 4B beads (Sigma-Aldrich). The sepharose 4B bead suspension was incubated on a rotator for 1 h at 4°C and centrifuged at 1000 g for 5 min at 4°C. The supernatant was transferred to prewashed (in lysis buffer) magnetic streptavidin-coupled Dynabeads and incubated for 1 h at 4°C on a rotator. The supernatant was removed from the Dynabeads using a magnetic DynaMag rack. The Dynabeads were washed twice in low salt wash buffer (20mM Tris-HCl (pH 7.4), 100 mM KCl, 0.1% Triton X-100), twice in high salt wash buffer (20 mM Tris-HCl (pH 7.4), 500 mM KCl, 0.1% Triton X-100), twice in low salt wash buffer and once in normal wash buffer (20mM Tris-HCl (pH 7.4), 150 mM KCl, 0.1% Triton X-100) before resuspended in elution buffer (50mM Tris-HCl, 150 mM NaCl, 1% SDS, 1mM biotin)). The lysate was mixed with 5x SDS-PAGE loading buffer, boiled for 5 min at 95°C and subsequently analyzed by immunoblotting.

Protein expression and purification

Bacteria were pre-cultured in 25 mL lysogeny (LB) medium (supplemented with kanamycin and chloramphenicol) and incubated on rotating shakers at 37°C overnight. Bacteria pre-cultures were transferred to 500 mL LB medium and grown at 30°C to an OD of 0.6. Protein expression was induced with 250 μL 1 M Isopropyl β-D-1-thiogalactopyranoside and bacteria were incubated overnight at 20°C. Bacteria were harvested by centrifugation and resuspended in 15 μL lysis buffer (50 mM Tris, 125 mM NaCl, 2 mM dithiothreitol (DTT) (Sigma-Aldrich), 1% Triton X-100 (Sigma-Aldrich), 30 µg/ml DNAse 1 (BioNordika) and one tablet of cOmplete protease inhibitor cocktail (Roche) per 100mL buffer). The resuspended pellet was frozen to -80°C. The bacterial suspension was thawed at 4°C and cleared by centrifugation (18.000 x g for 30 min at 4°C). The supernatant was collected and incubated with 400μL glutathione-sepharose 4B beads (GEHealthcare) for 1.5 hour at 4°C on a rotator. The beads were spun at 3.000 x g for 5 min at 4°C, the supernatant was discarded, and the beads were resuspended in 15 mL wash buffer (50 mM Tris, 125 mM NaCl, 2 mM DTT and 0.01% Triton X-100). The washing step was repeated three times. The washed beads were transferred to Bio-Spin® chromatography columns (Bio-Rad Laboratories, Inc. cat. #7326008) and washed in 1 mL wash buffer. Bead solution on the column was incubated with 2 µL 0.075U Thrombin (EMD Millipore, Novagen®) in 250 µL wash buffer overnight at 4°C on a rotator. PICK1 was eluded on ice and absorption at 280 nm was measured on a TECAN plate reader or a Thermo Scientific™ NanoDrop 2000c. The protein concentration was calculated using lambert beers law (εA280PICK1=32320(cm*mol/L)-1).

Alexa Fluor 488 C_5 maleimide (Invitrogen™) labelling of GST-PICK1 WT and the coding variants was prepared as above, but without addition DTT using 2mM tris (2-carboxyethyl) phosphine instead. Alexa Fluor 488 C_5 maleimide was added to the Bio-Spin® chromatography columns prior to Thrombin restriction. The columns were sealed and incubated on a rotator for 4-16 hours at 4 °C. The bead solution was

washed 5x with wash buffer removing unconjugated dye. Thrombin was added and protein was eluded as described.

Fluorescence polarization assay

Fluorescence polarization measurements were carried out as previously described, both for the saturation and competition assay (5, 6). In short, for the saturation assay increasing concentration of PICK1 WT and the coding variants with a final volume of 100 μL was added to Corning® 96-well half area black flat bottom polystyrene non-binding surface microplates with 5 μL of OrghDAT-C13 (final concentration of 20 nM). Incubated for 20 min on ice before measured. In short, for the competition assay, a fixed concentration of PICK1-WT, PICK1 R247H (non-saturating) and a fluorescent tracer (OrghDAT-C15) was used. The peptide was dissolved in wash buffer (50 mM Tris, 125 mM NaCl, 2 mM DTT and 0.01% Triton X-100) at a concentration of 2 mM, and a final assay concentration of maximally 1 mM was used. Following 15 mins of pre-incubation, increasing concentrations of unlabelled DATC15 peptide were added and incubated for an additional 20 mins on ice in a Corning® 96-well half area black flat bottom polystyrene nonbinding surface microplate. Fluorescence polarization was measured using an Omega POLARstar plate reader at 488 nm and 535 nm. The 13- and 15-residue peptides from the C-terminal region of the human dopamine transporter ((RG)EVRQFTLRHWLKV) were purchased from Schafer-N A/S with >95% purity.

Liposome preparation

The lipids were dissolved in chloroform, before addition of 2% DiD (w/w) for the liposome deformation assay. Lipids were dried while rotating using N_2-gas, creating a thin lipid film, followed by vacuum dehydration for at least 10 hours to remove residual chloroform. Lipids were rehydrated in a sterile 200 nM D-Sorbitol solution (pH 7.4) to

a final concentration of 0.5 or 1 mg/mL for the liposome deformation assay or TEM imaging, respectively. The rehydrated lipids were put through minimum 8 “freeze-thaw” cycles; frozen using liquid nitrogen and thawed in a 40-50°C water bath. The liposomes were extruded through a 19 mm polycarbonate Whatman™ Nuclepore™ Track-Etched membrane with a pore size of 1 µm using a LiposoFast liposome extruder (Avestin). The liposomes were extruded at least 15 times before being diluted 1:200 in sterile phosphate buffered saline (PBS) with a total salt concentration of 100mM (94mM NaCl, 3.1mM $Na2HPO_4$ and 0.9mM $NaH2PO_4$, pH 7.4). For TEM imaging liposomes were diluted 1:10 in PBS (pH 7.4).

Flow cytometry

Increasing concentrations of purified PICK1 WT and PICK1 coding variants were incubated with a fixed concentration of liposomes (~2.5 µg/mL, 2% DiD (w/w)) and left at room temperature (RT) for 1 h prior to acquisition. Fluorescence intensity for DiD and AF488 were detected by photomultiplier tubes (PMTs) after passing through 660/20 nm and 505LP, 530/30 nm light filters, respectively. DiD was excited by a 633 nm JDS Uniphase HeNe laser (17 mW) and AF488 by a Coherent Sapphire 488 nm, air cooled laser (20 mW). Events were triggered by fluorescence, using the 488 nm laser filtered through a 685 long pass filter, and 695/40 nm bandpass filter. The voltage applied to the photomultiplier tube detecting events was set to 500V with a threshold value of 200, defined as the highest gain allowing a maximum of 1 event/s when running a filtered phosphate buffered saline solution. The voltages applied to the detectors of AF488 and DiD fluorescence were kept constant throughout experiments at 335V and 465V, respectively. We recorded at least 100,000 events per condition for each experiment, with a constant number of events per condition within each experiment.

For fission analysis, we applied gates removing >99.9% of the DiD background noise, acquired when running samples containing protein diluted in PBS. The resulting data

were applied in a kernel density estimation with 512 equally sized bins on a log scale ranging from 0 to 5 (A.U.) and normalized to the population obtained from a sample only containing liposomes by subtraction. Data were analyzed using FlowLogic software (Inivai).

Proinsulin and insulin ELISA

High range rat proinsulin and insulin ELISA kit immunoassays (ALPCO™) were used to measure the proinsulin and insulin content in INS-1E lysates. Cells were seeded at a density of 250,000 cells/well in 12-well tissue culture plates (TPP®, Sigma-Aldrich) four days prior to cell lysis. Protocol provided by the manufacturer was followed. Briefly, the kit consists of a 96-well microplate pre-coated with a monoclonal antibody for pro-insulin or insulin. Lysates and HRP-conjugated secondary antibodies were added. Absorption was measured using an Omega POLARstar plate reader at 450nm and 590nm.

Light microscopy

LSM 510 settings; Alexa Fluor® 488 and YFP fluorescent signals were detected using a 488 nm argon laser. Alexa Fluor® 568 and Alexa Fluor® 647 fluorescent signals were detected using a 543 nm helium-neon laser and a 633 nm helium-neon laser, respectively. LSM 710 settings; Alexa Fluor® 488, Alexa flour® 568 and Alexa Fluor® 647 fluorescent signals were detected using a 488 nm argon laser, a solid state 561 nm laser and a helium-neon 633nm laser, respectively. .Channels were imaged separately

Co-localization analysis

To examine colocalization between PICK1 and different cellular markers, Van Steensel's cross-correlation function was used on the processed images with the JaCoP plugin in ImageJ (7). The Van Steensel's cross-correlation calculates the Pearson

cross-correlation as the signal from channel one (ex. Cyan PICK1) shift relative to the signal from channel two (ex. Magenta insulin) in the x-direction pixel per pixel. The peak at $\Delta x = 0$ indicates a specific colocalization and a value of 1 indicates total overlap. Analysis of colocalization was performed blinded.

dSTORM imaging

Images were acquired at 10,000-30.000 cycles of one frame of 561 nm laser activation followed by one frame of 647 nm laser activation at 16 Hz per cycle. Shutters regulated the light path in order to separate the light between the frames. The 561 nm and 647 nm lasers were held constant at 0.6 and 1.1 kW cm^{-2}, respectively, while a 405 nm laser was gradually increased to <0.1 kW cm^{-2}.

Airyscan 2 image analysis

Images detected with the Airyscan 2 detector were processed in the Zen software (Carl Zeiss, Oberkochen, Germany) using airyscan mode to generate a single image, same setting was used for each individual experiment. Due to high background noise in the 488 channel, untransduced β-cells were immunostained for eGFP and the highest intensity value observed was used as threshold to identify eGFP positive and negative cells for each individual experiment. ROIs in the 488-channel combined with the DAPI channel and insulin channel were used to identify cells. Prior to quantification of the immunosignal, an auto threshold (v1.17.2) from the ImageJ software was employed for each channel. These settings were held constant throughout each individual experiment. The images were converted to binary images and multiplied into each ROI of the original image, resulting in a total intensity value. For total immunosignal, the immunosignals from transduced cells were normalized to the mean of untransduced cells from each individual experiment. Quantification was performed blinded.

dSTORM image analysis

Localizations were fitted to the dSTORM movies with the ThunderSTORM plugin for imageJ (8). Images were filtered with the Wavelet filter (B-Spline), and localizations detected with the local maximum detector with a threshold of 2.0*std (Wave.F1). Drift was corrected with cross correlation and localization with uncertainty greater than 20 nm was filtered out. For the colocalization analysis, we used coordinate based colocalization (CBC) (9) as previously described for PICK1 and insulin clusters (1). The CBC was completed in 20 steps of 5nm, and the clusters were identified with the DBSCAN algorithm through the sklearn library in python 3.6, where a localization was classified as clustered if it had 10 localizations or more within a 40nm radius. Each localization within a PICK1 cluster was assigned a CBC value ranging from 1 to –1 based on the distance to clusters of insulin and vice versa. Values of 1 define a perfect overlap between a PICK1 and an insulin cluster, whereas a value of –1 indicates no overlap between the clusters. DBSCAN was performed in 3D. Diameter was determined from the 2D area of the convex hull of the cluster in the X-Y plane by fitting the area to a circle. The software programs ImageJ and MATLAB (MathWorkers, Natick, MA, USA) were used for image visualization.

SIM image analysis

Images were reconstructed using a built-in algorithm in the Zen software. A noise filter was added and held constant for each individual experiment, although always set between -4.2 and -4.5. Bead alignment matrix was used for drift correction. For 3D visualization we used the Amira software 2019.1 (Thermo Fisher Scientific, Waltham, MA, USA).

AlphaFold2 Setup

AlphaFold2 structures were predicted based on multiple sequence alignment results mapped using mmseqs2. 5 models were predicted for each model and ranked based

on their respective pTMscores. The structures were fed back into the neural network a maximum of 3 times for refinement. Subsequently, the sidechains of the refined structures were relaxed using Amber-Relax (10)

Reference

1. Herlo R, Lund VK, Lycas MD, Jansen AM, Khelashvili G, Andersen RC, et al. An Amphipathic Helix Directs Cellular Membrane Curvature Sensing and Function of the BAR Domain Protein PICK1. *Cell Rep.* 2018;23(7):2056-69.
2. Jansen AM, Nassel DR, Madsen KL, Jung AG, Gether U, and Kjaerulff O. PICK1 expression in the Drosophila central nervous system primarily occurs in the neuroendocrine system. *J Comp Neurol.* 2009;517(3):313-32.
3. Hewes RS, Park D, Gauthier SA, Schaefer AM, and Taghert PH. The bHLH protein Dimmed controls neuroendocrine cell differentiation in Drosophila. *Development.* 2003;130(9):1771-81.
4. Pietropaolo M, Castano L, Babu S, Buelow R, Kuo YL, Martin S, et al. Islet cell autoantigen 69 kD (ICA69). Molecular cloning and characterization of a novel diabetes-associated autoantigen. *J Clin Invest.* 1993;92(1):359-71.
5. Erlendsson S, Rathje M, Heidarsson PO, Poulsen FM, Madsen KL, Teilum K, et al. Protein interacting with C-kinase 1 (PICK1) binding promiscuity relies on unconventional PSD-95/discs-large/ZO-1 homology (PDZ) binding modes for nonclass II PDZ ligands. *JBC.* 2014;289(36):25327-40.
6. Madsen KL, Beuming T, Niv MY, Chang CW, Dev KK, Weinstein H, et al. Molecular determinants for the complex binding specificity of the PDZ domain in PICK1. *J Biol Chem.* 2005;280(21):20539-48.
7. Bolte S, and Cordelieres FP. A guided tour into subcellular colocalization analysis in light microscopy. *J Microsc.* 2006;224(Pt 3):213-32.
8. Ovesny M, Krizek P, Borkovec J, Svindrych Z, and Hagen GM. ThunderSTORM: a comprehensive ImageJ plug-in for PALM and STORM data analysis and super-resolution imaging. *Bioinformatics.* 2014;30(16):2389-90.
9. Malkusch S, and Heilemann M. Extracting quantitative information from single-molecule super-resolution imaging data with LAMA - LocAlization Microscopy Analyzer. *Sci Rep.* 2016;6:34486.
10. Jumper J, Evans R, Pritzel A, Green T, Figurnov M, Ronneberger O, et al. Highly accurate protein structure prediction with AlphaFold. *Nature.* 2021;596(7873):583-9.

Supplemental figures

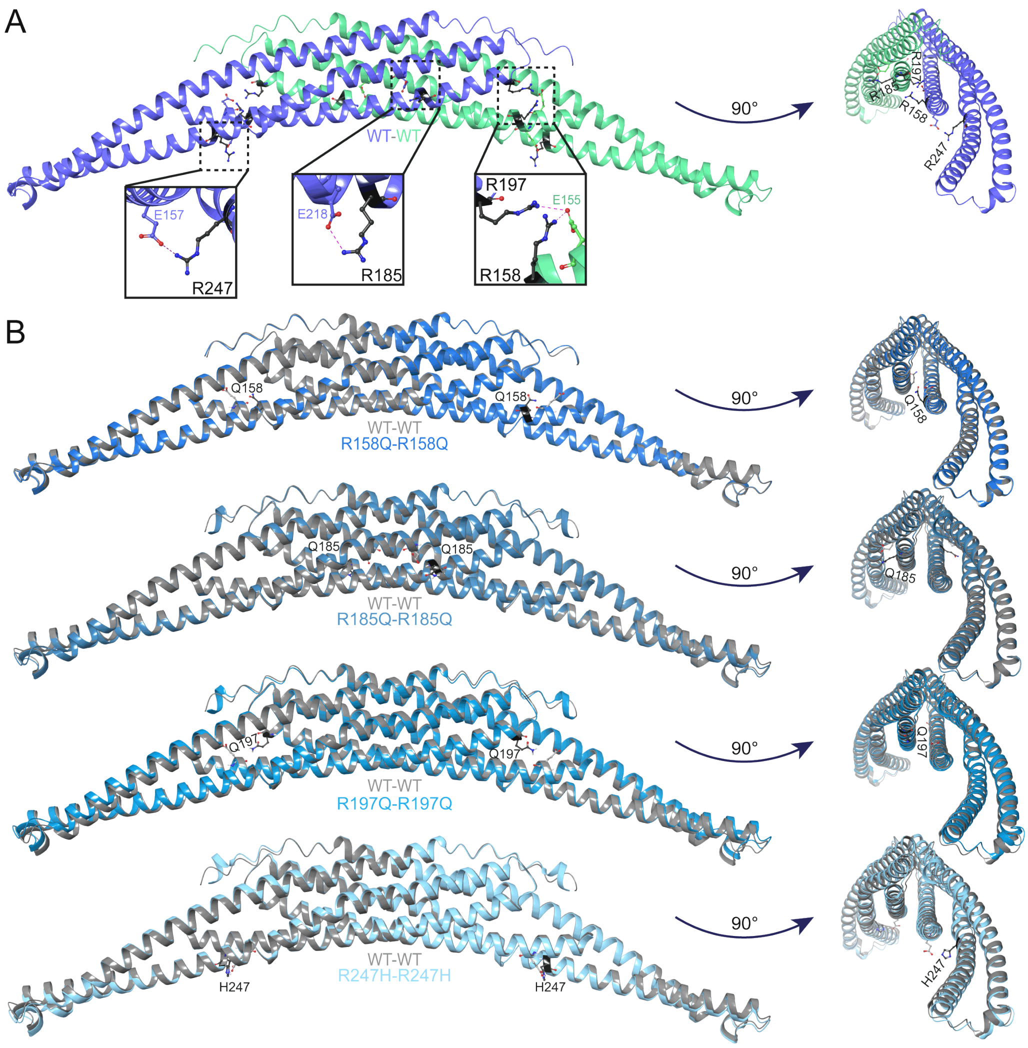

Supplemental Figure 1. Structure of the dimeric PICK1 BAR domain with the four coding variants. (A) Ribbon diagram showing the homodimeric complex of the PICK1 BAR domain (individual monomers represented as purple and green) (AlphaFold 2.0 Prediction, pLDDT: 76.18, pTMscore: 0.6451) and the localization of the four arginine's (carbon atoms: black, nitrogen atoms: blue) identified as coding variants. *Inserts* show the amino acid residues of the PICK1 coding variants as sticks with nitrogen atoms (blue) and oxygen atoms (red). Putative charge-charge interactions with nearby negatively charged residues (carbon atoms: purple or green) are shown with pink dashed lines (distances 3.08Å). 90° rotation of homodimeric PICK1 BAR domain; labels

annotate the arginines substituted in each of the coding variants. (B) Superimposition of the homodimeric BAR domain of PICK1 WT (grey) with the homodimers of PICK1 coding variants R158Q, R185Q, R197Q and R247H (shades of blue). AlphaFold 2.0 Predictions: R158Q (pLDDT: 76.50, pTMscore: 0.6445), R185Q (pLDDT: 75.78, pTM-score: 0.6376), R197Q (pLDDT: 76.18, pTMscore: 0.6452), R247H (pLDDT: 75.96, pTMscore: 0.6368). 90° rotation: labels show the amino acid substitution in each coding variants.

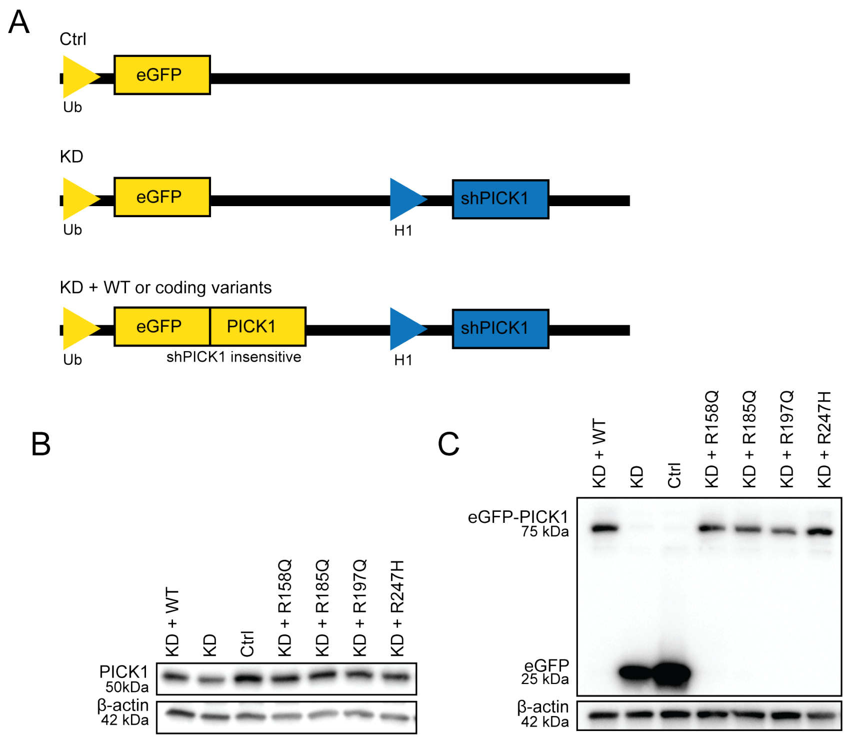

Supplemental Figure 2. Immunoblotting shows reduced level of endogenous PICK1 with an expression of GFP or GFP-PICK1 in INS-1E cells transduced with the lentiviral constructs. (A) Schematic diagram of the different FUGW constructs; ctrl, KD, and KD + WT or KD + coding variants. (B) Immunoblotting of INS-1E lysates show expression of endogenous PICK1, and (C) GFP(-PICK1). Note that low transduction efficiency result in only weak knock down as assessed in bulk by western blotting. Knock down in individual transduced cells as assessed by immunocytochemistry was much more robust as seen in figure 2.

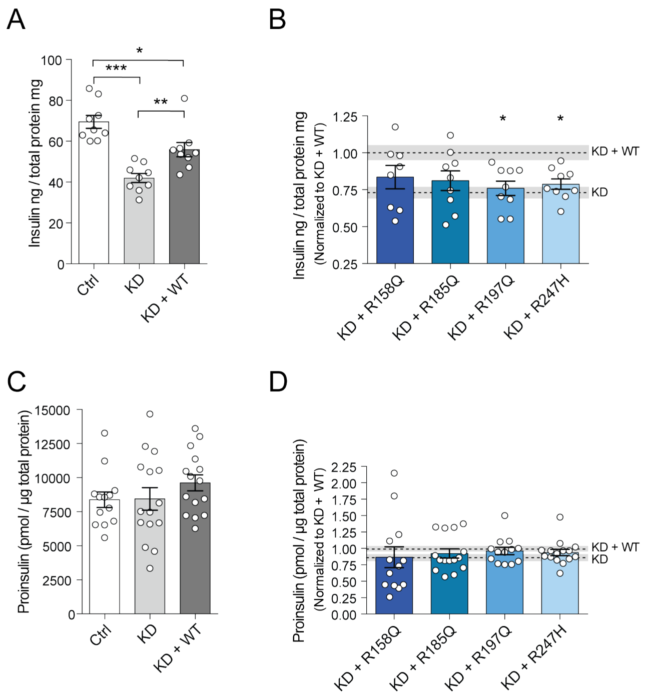

Supplemental Figure 3. R197Q and R247H compromise PICK1 function in insulin storage but not proinsulin content in INS-1E cells as assessed in bulk by ELISA assay. Sandwich ELISA was performed on lysates from transduced INS-1E cells. Data are shown as bar plot with dots and mean ± SEM with one-way ANOVA followed by Tukey's or Dunnett's multiple comparisons test if nothing else is stated. (A) Quantification of the insulin content in INS-1E cells expressing ctrl, KD and KD + WT, n = 8-9 samples pr. genotype. (B) Quantification of the insulin content from INS-1E cells expressing the four PICK1 coding variants, normalized to the mean insulin content from KD + WT from (A). The two dotted lines represent mean ± SEM from KD and KD + WT from (A). n = 8-9 samples pr. genotype. (C) Quantification of the proinsulin content from INS-1E cells expressing ctrl, KD or KD + WT, n = 13-15 samples pr. genotype. (D) Quantification of the proinsulin content from INS-1E cells expressing the four PICK1 coding variants, normalized to the mean proinsulin content from KD + WT from (D). Data are shown as bar plot with dots and mean ± SEM with Kruskal Wallis test. The two dotted lines represent mean ± SEM from KD and KD + WT from (C), n = 13-15 samples pr. genotype. (*p<0.05, **p<0.01, ***p<0.001).

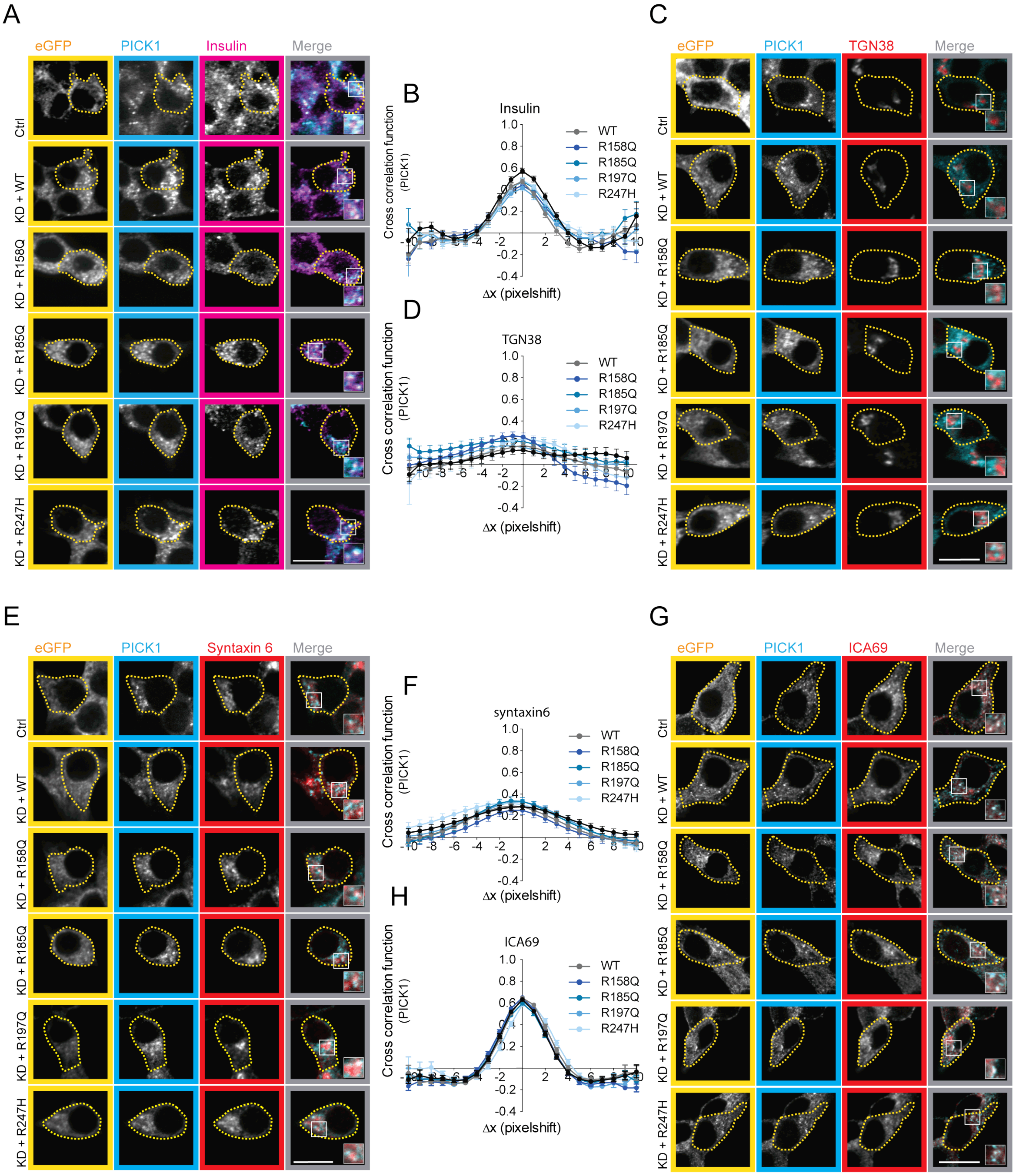

Supplemental Figure 4. No change in the localization of PICK1 in the RSP with the four PICK1 coding variants. Representative confocal images of INS-1E cells transduced with the lentiviral constructs and immunostained for eGFP (yellow), PICK1 (cyan), insulin (magenta), TGN38 (red), syntaxin 6 (red) or ICA69(red). Transduced cells (eGFP-positive) are outlined with a yellow dotted line. Scale bar = 10 μm. Merged

images are with inserts highlighting areas of overlapping localization. Zoom (50%) is gain adjusted for visualization. The colocalization was quantified by the Van Steensel's cross-correlation function. (A) Confocal images of INS-1E cells immunostained for eGFP, PICK1, and insulin. (B) Colocalization between PICK1 and insulin, ctrl (endogenous PICK1) (n =69), KD + WT (n = 64), KD + R158Q (n = 54), KD + R185Q (n = 64), KD + R197Q (n = 57), and KD + R247H (n = 58). (C) Confocal images of INS-1E cells immunostained for eGFP, PICK1 and TGN38 (marker for TGN). (D) Colocalization between PICK1 and TGN38. KD+ R158Q (n = 31), KD + R185Q (n = 31), KD + R197Q (n = 27), KD + R247H (n = 20), ctrl (n = 27) and KD + WT (n = 39 (E) Confocal images of INS-1E cells immunostained for eGFP, PICK1 and syntaxin 6 (marker for TGN and early ISGs). (F) Colocalization between PICK1 and syntaxin 6. KD+ R158Q (n = 26), KD + R185Q (n = 34), KD + R197Q (n = 28), KD + R247H (n = 27), ctrl (n = 37) and KD + WT (n = 34). (G) Confocal images of INS-1E cells immunostained for eGFP, PICK1 and ICA69 (H) Colocalization between PICK1 and ICA69 KD+ R158Q (n = 46), KD + R185Q (n = 52), KD + R197Q (n = 45), KD + R247H (n = 46), ctrl (n = 54) and KD + WT (n = 54). Data are shown as mean ± SEM.

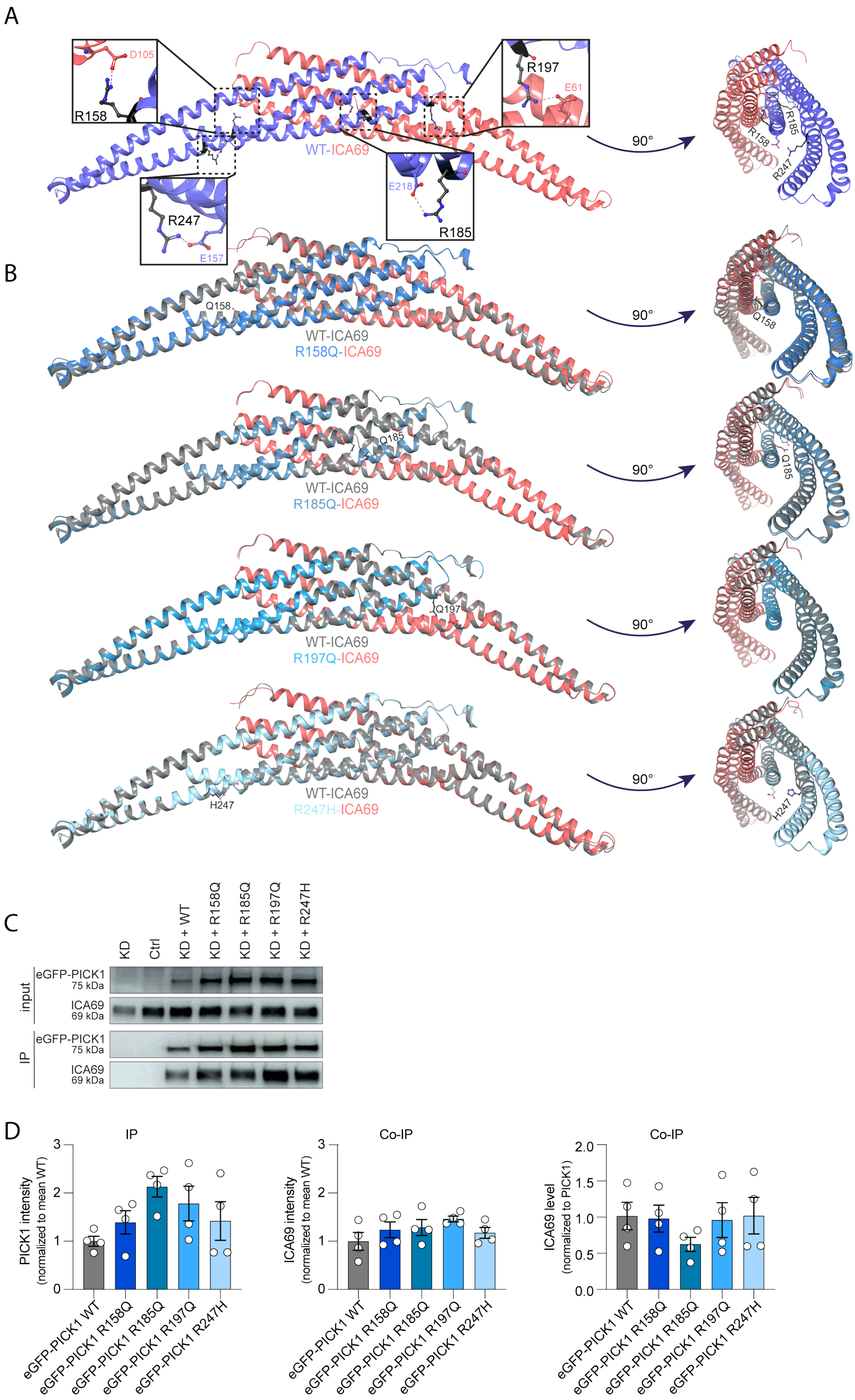

A
D105
R158
R197
E61
WT-ICA69
E218
R185
R247
E157
90°
R185
R158
R247
B
Q158
WT-ICA69
R158Q-ICA69
90°
Q158
Q185
WT-ICA69
R185Q-ICA69
90°
Q185
Q197
WT-ICA69
R197Q-ICA69
90°
H247
WT-ICA69
R247H-ICA69
90°
H247
C
KD
Ctrl
KD + WT
KD + R158Q
KD + R185Q
KD + R197Q
KD + R247H
input
eGFP-PICK1 75 kDa
ICA69 69 kDa
IP
eGFP-PICK1 75 kDa
ICA69 69 kDa
D
IP
PICK1 intensity (normalized to mean WT)
Co-IP
ICA69 intensity (normalized to mean WT)
Co-IP
ICA69 level (normalized to PICK1)
eGFP-PICK1 WT
eGFP-PICK1 R158Q
eGFP-PICK1 R185Q
eGFP-PICK1 R197Q
eGFP-PICK1 R247H

Supplemental Figure 5. Structure of the heterodimeric PICK1-ICA69 BAR domains with the four coding variants. (A) Ribbon diagram showing the heterodimeric complex of the PICK1-ICA69 BAR domains (PICK1WT: purple, ICA69: red) (AlphaFold 2.0 Prediction, pLDDT:69.42 pTMscore:0.6357) and the localization of the four arginine's (carbon atoms: black, nitrogen atoms: blue) identified as coding variants. *Inserts* show the amino acid residues of the PICK1 coding variants as sticks with nitrogen atoms (blue) and oxygen atoms (red). Putative charge-charge interactions with nearby negatively charged residues (carbon atoms: purple or red) are shown with pink dashed lines (distances 3.08Å). 90° rotation of heterodimeric PICK1-ICA69 BAR domains: labels annotate the arginines substituted in each of the coding variants. (B) Superimposition of the heterodimeric PICK1-ICA69 BAR domain complex (grey) with the heterodimeric ICA69 (red) complexes of PICK1 coding variants R158Q, R185Q, R197Q and R247H (shades of blue). AlphaFold 2.0 Predictions: R158Q (pLDDT:70.02 pTMscore:0.6412), R185Q (pLDDT:70.10 pTMscore:0.6398), R197Q (pLDDT:69.67 pTMscore:0.6326), R247H (pLDDT:69.85 pTMscore:0.6388). 90° rotations: labels show the amino acid substitution in each coding variants. (C) Representative immunoblots of immunoprecipitated and input lysates from transduced INS-1E cells. Upper two panels represent input expression of eGFP-PICK1 and ICA69 and lower two panels represent IP and Co-IP expression of eGFP-PICK1 and ICA69, respectively. (D) Quantified expression levels of PICK1 IP, ICA69 Co-IP and ICA69 Co-IP normalized to PICK1 IP. Data are shown as bar plot with dots and mean ± SEM with one-way ANOVA followed by either Dunnett's multiple comparisons test for statistical comparison. n = 4 individual experiments.

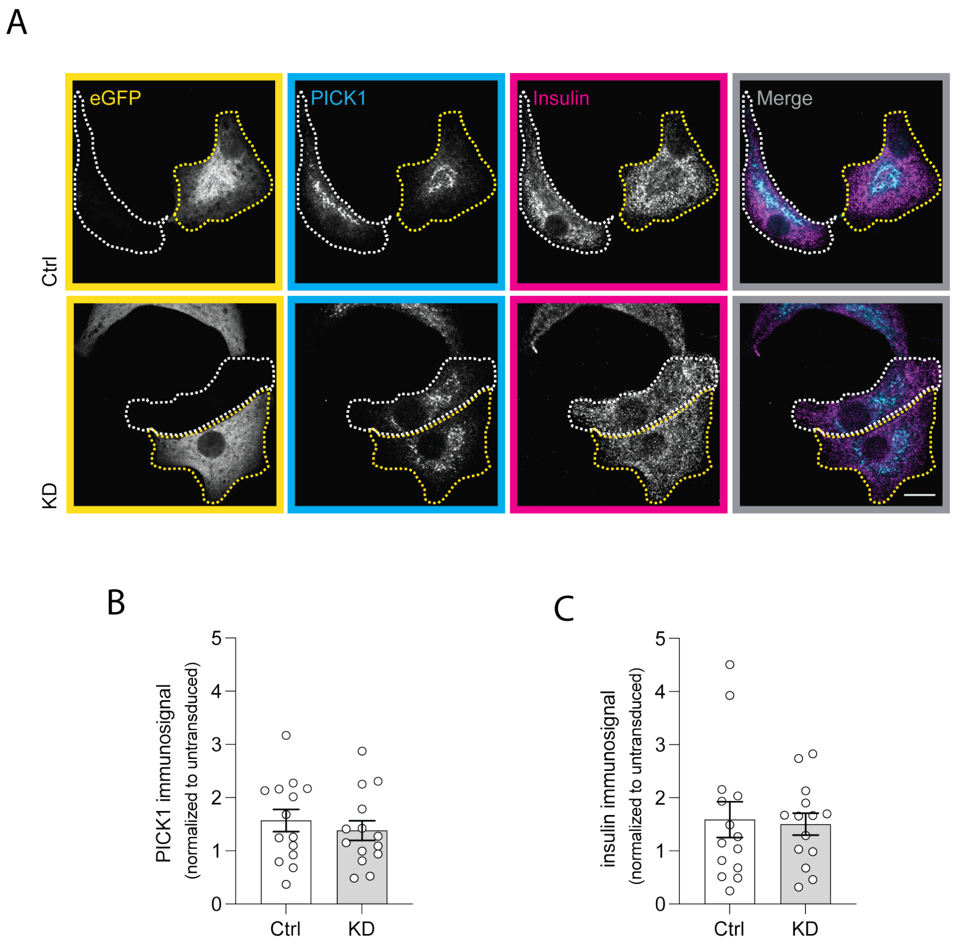

Supplemental Figure 6. The lentiviral shRNA rPICK1 vector shows no KD of PICK1 in mouse β-cells. (A) Representative confocal images of mouse β-cells transduced with the lentiviral constructs ctrl and KD, and immunostained for eGFP (yellow), PICK1 (cyan) and insulin (magenta). The merged images show the PICK1 and insulin immunosignals. Examples of transduced cells (eGFP-positive) are outlined with yellow dotted lines and untransduced cells are outlined with white dotted lines. Scale bar = 10 µm. (B) Quantification of the PICK1 immunosignal. KD (n = 14) compared to ctrl (n =14) with an unpaired t-test. (C) Quantification of the insulin immunosignal. KD compared to ctrl using the Mann-Whitney test.

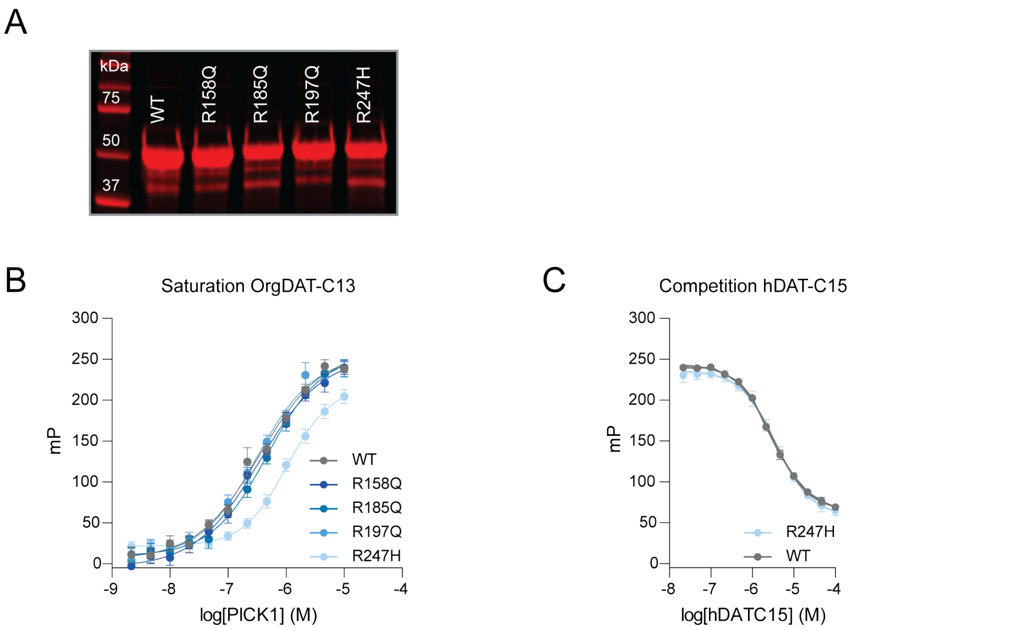

Kd	
WT 0.35 µm [0.25 - 0.55]	R197Q 0.31 µm [0.23 - 0.45]
R158Q 0.34 µm [0.25 - 0.54]	R247H 1.03 µm [0.80 - 1.48]
R185Q 0.48 µm [0.35 - 0.70]	

Ki	
WT 1.05 µm [0.92 - 1.20]	R247H 1.15 µm [0.74 - 1.81]

Supplemental Figure 7. Expression and PDZ binding ability for purified PICK1 WT and coding variants. (A) Representative SDS-page shows the expression level of purified full-length PICK1 WT, R158Q, R185Q, R197Q and R247H, respectively. (B) Binding of Org-hDATC13 to the PDZ domain in purified PICK1 WT, R158Q, R185Q, R197Q and R247H determined by fluorescent polarization (FP) saturation binding with affinities (K_D) listed below. Data represented for n = 3 and shown as mean ± SEM. (E) FP competition binding of PICK1 WT and R247H competing for binding to hDAT-C15 against Org-hDAT-C13, with affinity (K_i) listed below. n = 6, data shown as mean ± SEM.

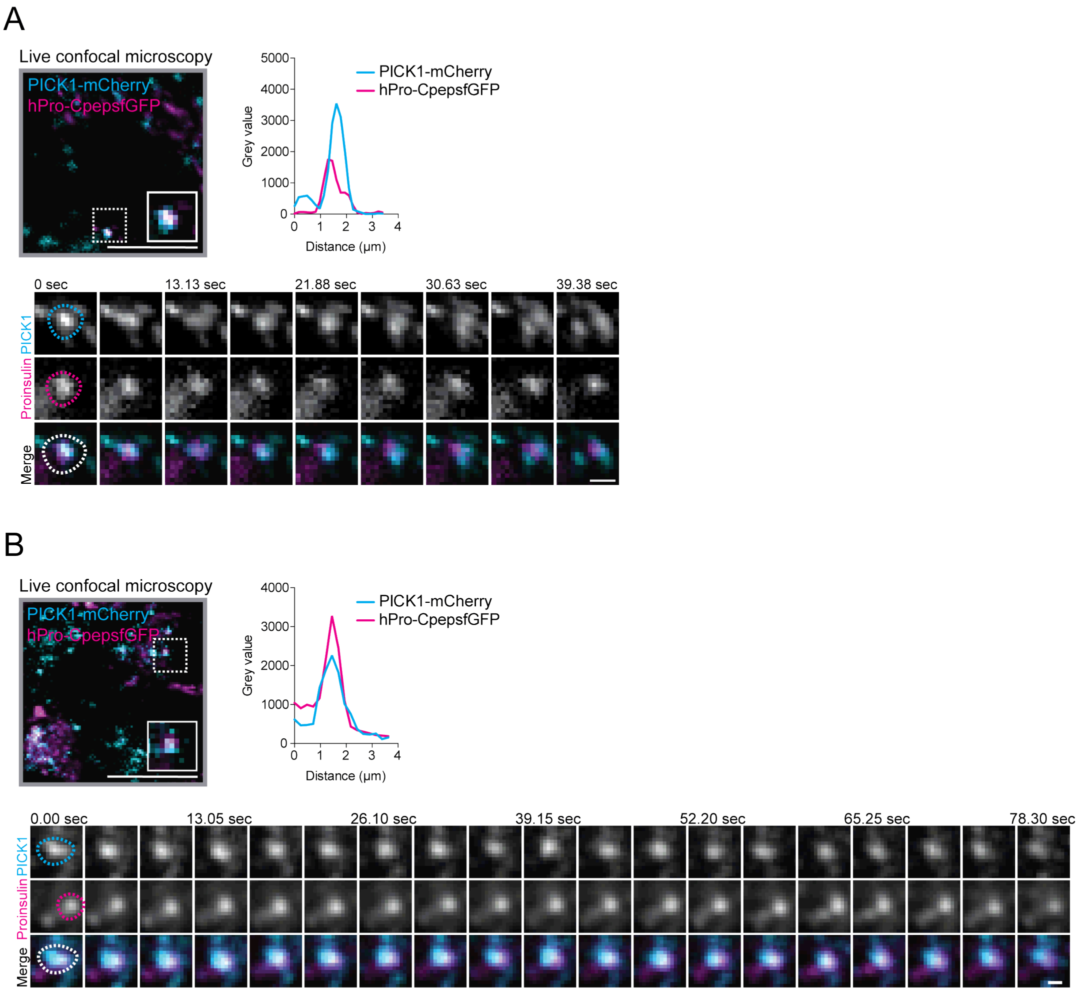

Supplemental Figure 8. PICK1-positive clusters interact with proinsulin clusters. GRINCH cells were transiently transfected with PICK1-mCherry. (A) and (B) merged images of PICK1-mCherry (cyan) and hPro-CpepsfGFP (magenta) with inserts (50% zoom) highlighting areas of overlapping localization. Scale bar = 10 µm. Right shows profile plots from the inserts. Representative merged z-stack (500 nm confocal slice) images of PICK1 clusters and hPro-CpepsfGFP clusters overlapping during live microscopy under steady-state conditions (11mM glucose). Time-lapse imaging was of two-color z-stacks, acquired every 4.34 seconds. Upper two rows represent a PICK1-mCherry positive cluster and a hPro-CpepsfGFP cluster, respectively, both shown in greyscale. The third row shows merged images. Time is in seconds and scale bar = 1 µm.

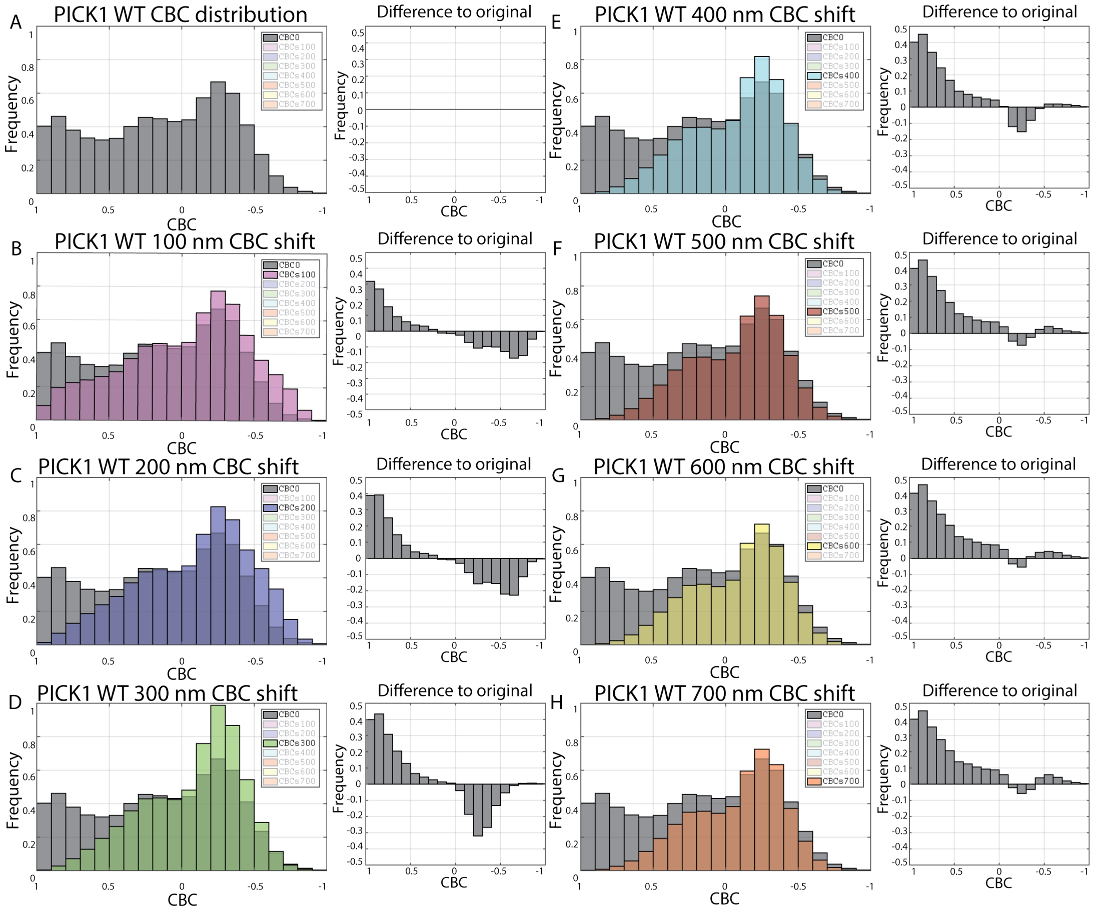

Supplemental Figure 9. Insulin CBC shift analysis to eliminate random colocalization. (A) The CBC distribution of PICK1 localization to insulin granules in INS-1E cells transduced with KD + WT. (B-H) The PICK1 clusters were shifted +100 nm per interval in the x direction, and the CBC distribution of PICK1 localization to the insulin granules are recalculated and compared with the CBC distribution from (A) (grey). Right panels show the difference in CBC between (A) and (B-H), which we refer to as random proximity subtracted CBC (rpsCBC) distribution. The analysis represents 3 analyzed images. Note that many points are not assigned a CBC values (NA) when shifted and this proportion increases with distance of the shift. The 'difference to original' plot show no further development beyond 500 nm.

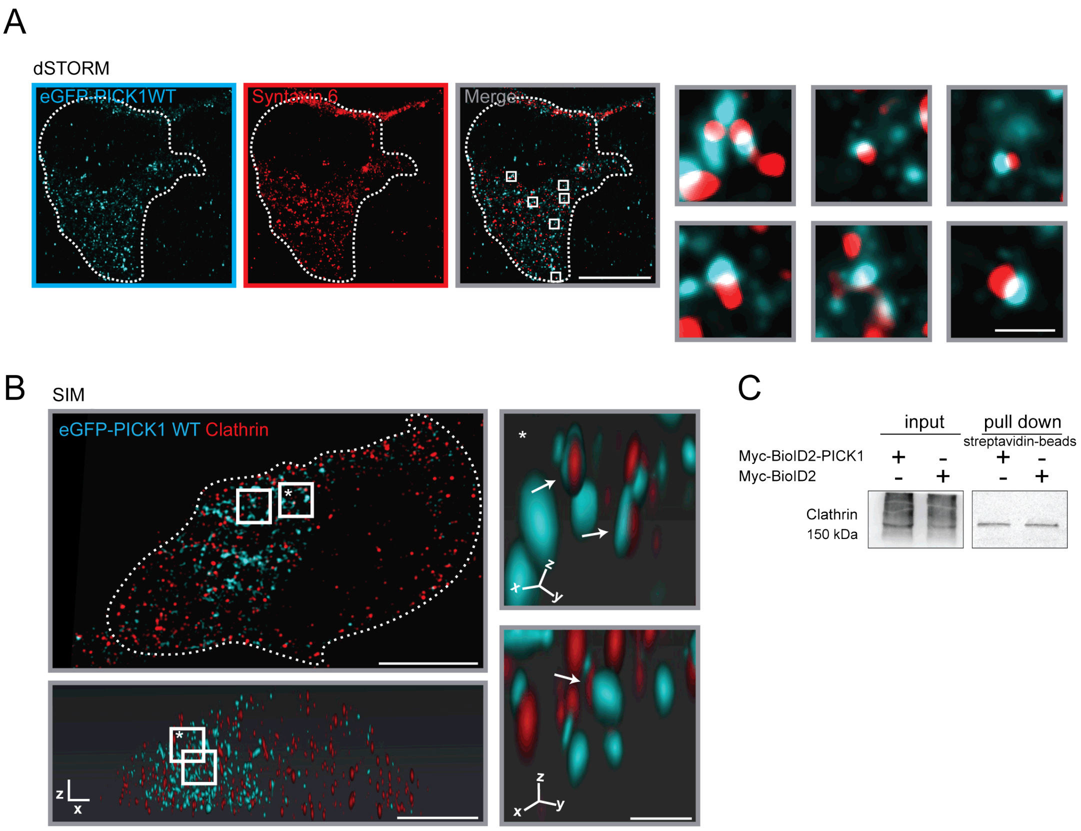

Supplemental Figure 10. Syntaxin 6 and clathrin colocalization with PICK1

(A) Representative dSTORM images of INS-1E cells transduced with KD + WT, and immunostained for eGFP-PICK1 (cyan) and syntaxin 6 (red). Scale bar = 5 µm. Right shows insert with higher magnification of small overlapping PICK1 and syntaxin 6 clusters (<150 nm). Scale bar = 250 nm. (B) Representative SIM image of INS-1E cells transduced with KD + WT, and immunostained for eGFP-PICK1 (cyan) and clathrin (red). Scale bar = 5 µm. Bottom shows same INS-1E cell in 3D reconstruction, and right shows insert with higher magnification of overlapping PICK1 and clathrin clusters (arrows). Scale bar = 500 nm. (C) INS-1E cells were transiently transfected with a Myc-BioID2-PICK1 construct or as control with Myc-BioID2. Biotinylated proteins were pulled down from cell lysates with streptavidin-beads for immunoblotting against clathrin. n = 4 individual experiments.

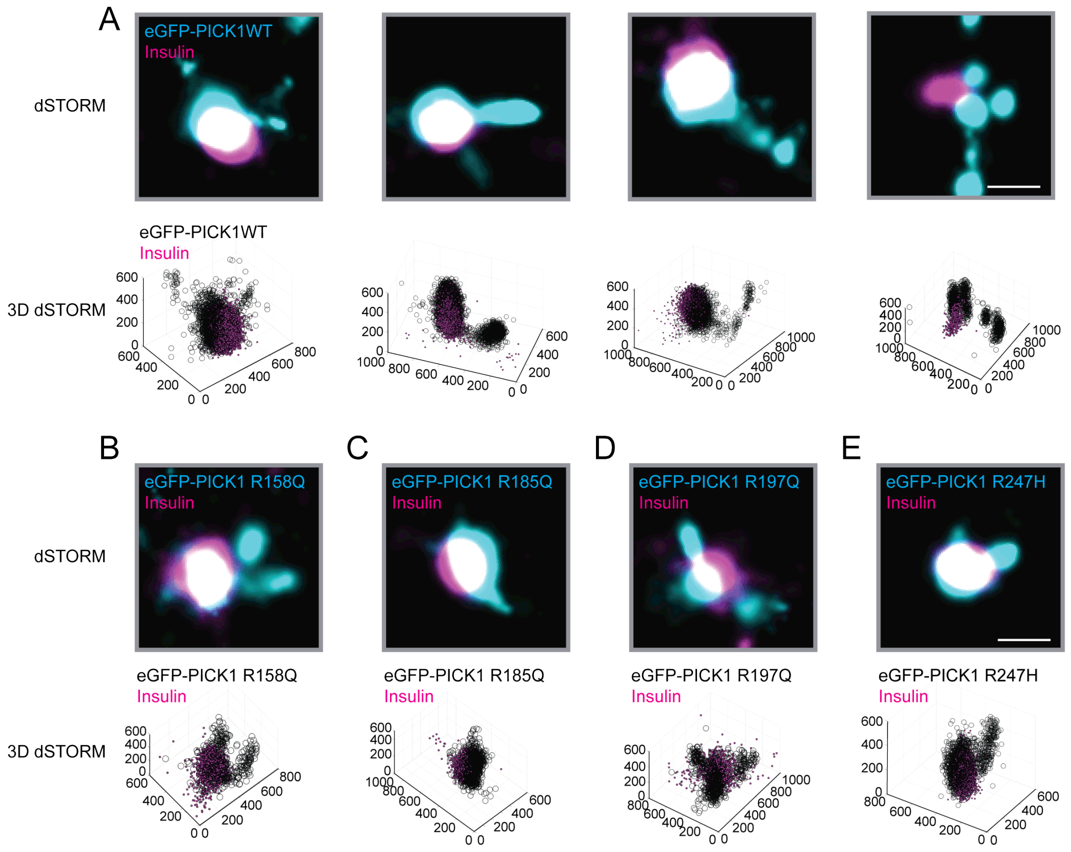

Supplemental Figure 11. PICK1 WT and the coding variants localize to extensions from insulin granules. INS-1E cells transduced with the lentiviral constructs and immunostained for eGFP-PICK1 and insulin. Upper row show representative dSTORM images of an insulin granule (magenta) colocalized with PICK1 (cyan) which is also extending from the granule. Scale bar = 250 nm. The lower row shows the 3D structure of the insulin granule (magenta) and PICK1 (black). Axis are in nm. (A) INS-1E cells transduced with the lentiviral construct KD + WT. (B to E) INS-1E cells transduced with the PICK1 coding variants.

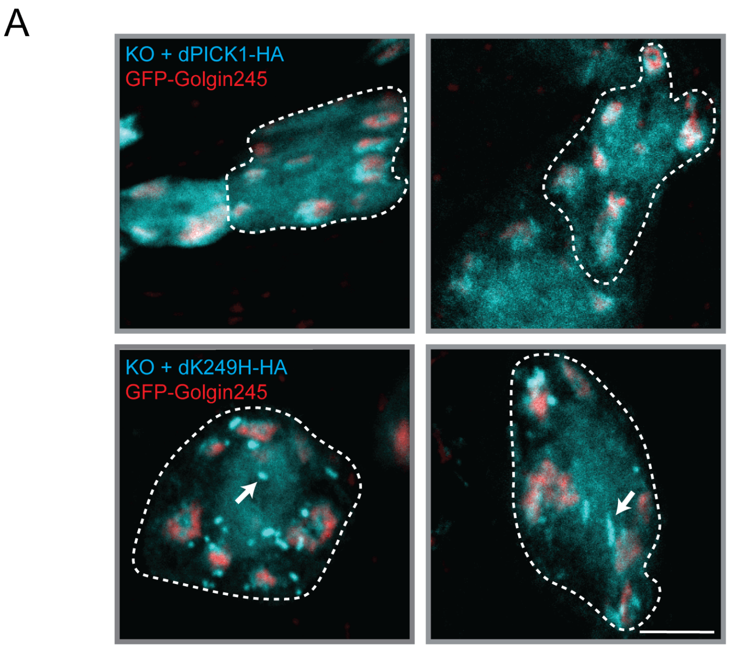

Supplemental Figure 12. Perturbed subcellular distribution of *Drosophila* PICK1^{K249H} in peptidergic neurons. (A) Anti-HA immunolabeling of large peptidergic somata in the ventral nerve cord of pupal flies expressing the TGN marker GFP-Golgin245 (red) and either WT dPICK1-HA (top) or dPICK1K249H-HA (bottom) (cyan) in a dPICK1 null background. White dotted lines represent peptidergic neurons. dPICK1K249H-HA forms brightly labeled punctate clusters and tubules (arrows) in the cytosol that are not seen for WT dPICK1-HA. These structures remain tightly associated with the TGN. Scale bar = 5 µm.

Full unedited blot for Figure 3: COS7 cells transfected with PICK1 constructs

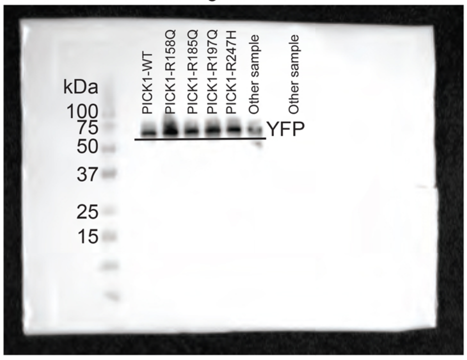

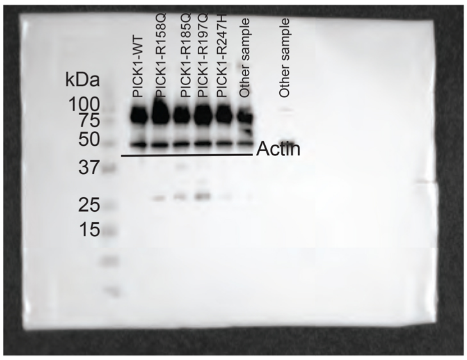

Full unedited blot for Figure 9: INS-1E cells transfected Myc-BioID2PICK1 or Myc-BioID2 and biotinylated proteins were pulled down with Dynabeads

input

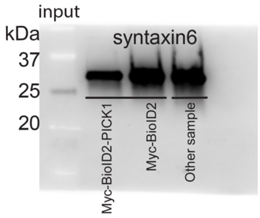

pull down

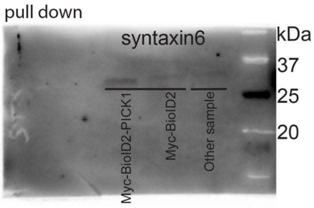

Full unedited blot for Supplemental Figure 2: INS-1E cells transduced with lentiviral constructs

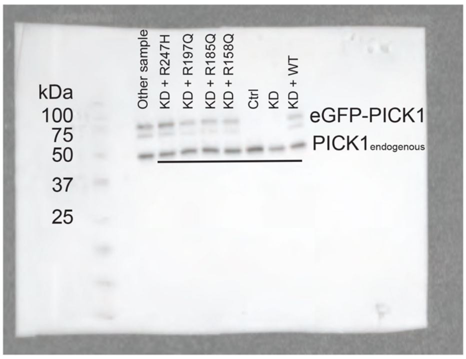

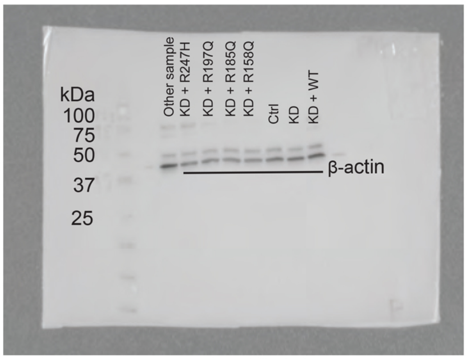

Full unedited blot for Supplemental Figure 2: INS-1E cells transduced with lentiviral constructs

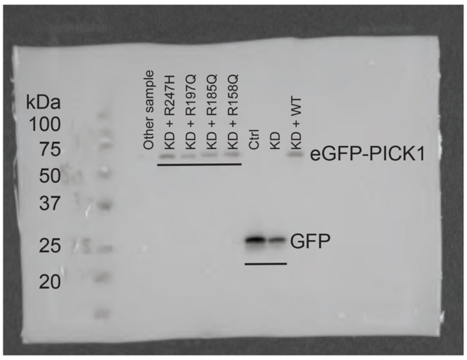

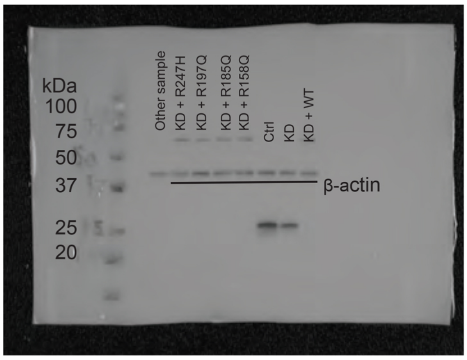

Full unedited blot for Supplemental Figure 5: INS-1E cells transduced with lentiviral constructs and immunoprecipitated with GFP trap particles

input

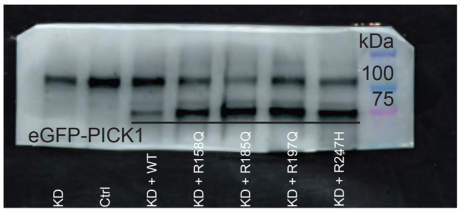

input

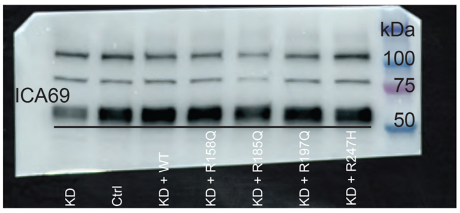

IP

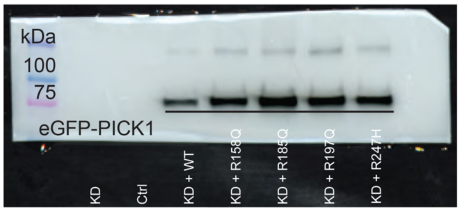

IP

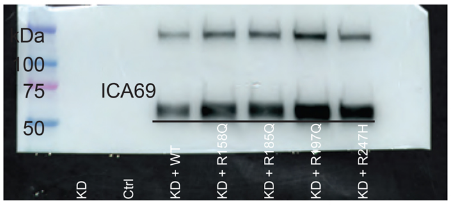

Full unedited blot for Supplemental Figure 10: INS-1E cells transfected Myc-BioID2PICK1 or Myc-BioID2 and biotinylated proteins were pulled down with Dynabeads

input

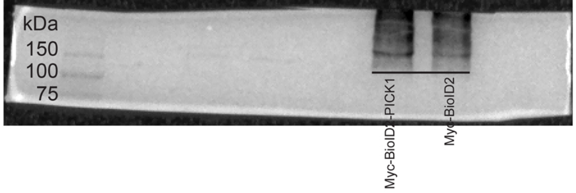

pull down

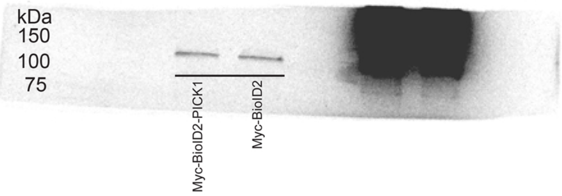

Full unedited gel for Supplemental Figure 7: Bacterial purified PICK1 and coding variants

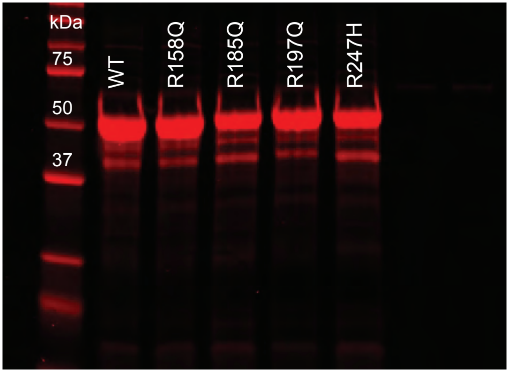

Supplement: Supplemental data [file jci-132-144904-s159.pdf]
